# Rutin interferes with hnRNP L phase separation and modulates the PKM2–lactate–H3K9la axis in pulmonary fibrosis

**DOI:** 10.3389/fphar.2026.1787570

**Published:** 2026-10-16

**Authors:** Jianlin Luan, Yujie Wang, Mengqi Jiang, Haitong Zhang, Changjun Lv, Jinjin Zhang, Xiaodong Song

**Affiliations:** 1 School of Pharmacy, Shandong Key Lab of Complex Medical Intelligence and Aging, Shandong Medical and Pharmaceutical University, Yantai, China; 2 Department of Respiratory and Critical Care Medicine, Shandong Medical and Pharmaceutical University Hospital, Shandong Medical and Pharmaceutical University, Binzhou, China

**Keywords:** pulmonary fibrosis, rutin, hnRNP L, phase separation, H3K9la, PKM2

## Abstract

**Background:**

Pulmonary fibrosis is a chronic and progressive lung disease with limited treatment options; it is urgent to develop new drugs.

**Purpose:**

This study investigated the anti-pulmonary fibrosis effects and mechanism of rutin, and sought to provide a potential lead compound for further development of anti-pulmonary fibrosis agents.

**Methods:**

The anti-pulmonary fibrosis effect of rutin was assessed using Cell Counting Kit-8 (CCK-8), real-time cell proliferation analysis and scratch wound healing assay in Transforming Growth Factor-β1(TGF-β1)-stimulated Medical Research Council cell strain-5 (MRC-5) cells, along with lung function tests, MicroCT imaging, Hematoxylin and Eosin (H&E) and Masson staining in mice with induced pulmonary fibrosis. The mechanism of rutin was explored through molecular docking, the surface plasmon resonance assay, the cell thermal shift assay (CETSA), droplet formation observation, fluorescence recovery after photobleaching, transcriptome sequencing, Enzyme Linked Immunosorbent Assay (ELISA), Co-immunoprecipitation (Co-IP) and Chromatin Immunoprecipitation-Polymerase Chain Reaction (ChIP-PCR).

**Results:**

Rutin inhibited fibroblast-to-myofibroblast differentiation, myofibroblast proliferation and migration, and extracellular matrix deposition. Moreover, rutin effectively improved lung function, including forced vital capacity, dynamic lung compliance and total lung capacity. Additionally, rutin markedly alleviated fibrotic features, such as honeycomb lung reduction, preserved alveolar structure and thinned alveolar walls. Mechanistically, our data support a model in which rutin may interfere with hnRNP L phase separation, and this interference is associated with reduced PKM2 generation, decreased lactate production and subsequent loss of H3K9la. The reduced H3K9la, in turn, correlated with suppressed PKM2 transcription, suggesting a self-limiting regulatory circuit.

**Conclusion:**

Our findings suggest that rutin may interfere with the regulatory interplay involving hnRNP L phase separation, PKM2 alternative splicing, lactate, and H3K9la, which is associated with the attenuation of pulmonary fibrosis. While these data reveal a potential mechanistic framework, definitive causality at each step of this regulatory axis remains to be established. Further studies are warranted to validate this model and to optimize the bioavailability of rutin for therapeutic application.

## 1 Introduction

Pulmonary fibrosis is a chronic, progressive interstitial lung disease with a steadily rising incidence and a median survival of only 3–5 years after diagnosis (Li C. et al., 2024). Its hallmark histopathological feature is the aberrant activation and differentiation of fibroblasts into myofibroblasts (Li et al., 2023). These cells deposit excessive extracellular matrix, progressively remodel lung architecture, and cause irreversible impairment of respiratory function (Allen et al., 2023). In recent years, the incidence of pulmonary fibrosis has increased annually (Pergolizzi et al., 2023). Unfortunately, current therapeutic options remain extremely limited (Mao et al., 2025). At present, only two antifibrotic drugs, nintedanib and pirfenidone, are approved for clinical use. Although these agents can modestly slow the decline in forced vital capacity, they fail to halt or reverse disease progression and are accompanied by prominent gastrointestinal side effects (Raghu and Fleming, 2024; Chianese et al., 2024). Therefore, there is an urgent need to identify new bioactive compounds and explore their mechanisms of action, providing a solid foundation for the development of effective therapeutic agents.

Natural products have emerged as promising sources of antifibrotic agents, as illustrated by Lepidium sativum seed extract, which attenuates pulmonary fibrosis by modulating the ncNRFR/let-7d regulatory pathway (Alanazi et al., 2025). Among these, flavonoids have recently gained particular attention due to their multi-targeting properties and favorable safety profiles. Rutin, a flavonol glycoside abundantly present in various plants, exhibits a broad range of pharmacological activities, including anti-inflammatory, antioxidant, and antitumor effects (Liu C. et al., 2024; Li D. et al., 2024). Notably, recent studies have demonstrated that rutin attenuates bleomycin-induced lung fibrosis in mice and inhibits TGF-β1-induced myofibroblast differentiation in lung fibroblasts, suggesting that rutin possesses anti-pulmonary fibrotic activity (Karunarathne et al., 2024). Despite these observations, the direct molecular target of rutin and the precise mechanism by which it exerts its anti-fibrotic effects—beyond its well-recognized antioxidant and anti-inflammatory actions—remain largely unknown.

Alternative pre-mRNA splicing is controlled by RNA-binding proteins called splicing factors, which bind specific sequence motifs to regulate exon inclusion (Lee and Rio, 2015). Heterogeneous nuclear ribonucleoprotein L (hnRNP L) is one such splicing factor that regulates transcripts involved in cell survival, proliferation, and differentiation (Zhou et al., 2022), and it promotes pulmonary fibrosis through circRNA back-splicing (Xu et al., 2022). Whether hnRNP L can be directly targeted by small-molecule drugs is unknown. Notably, drug repurposing efforts have shown that targeting RNA regulatory networks is feasible in pulmonary fibrosis: trimetazidine, an anti-anginal drug, alleviates bleomycin-induced fibrosis via the lncRNA CBR3-AS1/miRNA-29/FIZZ1 axis (Alzahrani et al., 2024).

Many RNA-binding proteins, including splicing factors, exert their functions through liquid–liquid phase separation (LLPS), forming dynamic biomolecular condensates (Chen et al., 2025; Licheva et al., 2025). Sequence analysis reveals that hnRNP L contains intrinsically disordered regions (IDRs), which are structural hallmarks of phase-separation-prone proteins. Dysregulated LLPS has been linked to various diseases, but whether hnRNP L undergoes LLPS during pulmonary fibrogenesis and can be pharmacologically targeted remains unexplored.

Given that hnRNP L is a splicing factor, we asked whether it regulates the alternative splicing of pyruvate kinase M (PKM), a key glycolytic enzyme. The PKM2 isoform is a rate-limiting glycolytic enzyme that promotes myofibroblast differentiation and pulmonary fibrosis progression. Enhanced PKM2-dependent glycolysis elevates lactate levels, providing substrate for histone lactylation. Notably, histone lactylation, including H3K9la, has been implicated in fibrotic remodeling, although its upstream regulation in lung fibroblasts remains incompletely understood (Li et al., 2026). Furthermore, PKM2 expression and activity in lung fibroblasts are also regulated by matrix stiffness, establishing a self-reinforcing cycle of fibroblast activation and tissue stiffening (Wang Y. et al., 2026). However, whether PKM alternative splicing is controlled by hnRNP L phase separation, and whether this connection constitutes a druggable axis in pulmonary fibrosis, has not been reported.

Based on the above evidence, we hypothesized that rutin directly binds hnRNP L, inhibits its phase separation, and thereby disrupts this metabolic–epigenetic feedback loop to alleviate pulmonary fibrosis. We tested this hypothesis using molecular docking, surface plasmon resonance, phase separation assays, transcriptome sequencing, and both *in vitro* and *in vivo* pulmonary fibrosis models. Building on our previous finding that hnRNP L phase separation promotes fibroblast-to-myofibroblast differentiation (Wang M. et al., 2026), the present study identifies rutin binds hnRNP L and interferes with its phase separation, and further links this event to the PKM2 alternative splicing–lactate–H3K9la regulatory circuit. These findings reveal a previously unappreciated connection between hnRNP L phase separation and metabolic–epigenetic regulation, which can be pharmacologically targeted by rutin.

Nevertheless, before detailing the mechanistic investigation, it is important to acknowledge a notable limitation of rutin—its poor oral bioavailability, which results from low aqueous solubility, limited intestinal absorption, and rapid metabolism. This pharmacokinetic drawback has long hindered its clinical translation despite its promising *in vitro* bioactivity. In the present study, we therefore used rutin as a tool compound to dissect a previously unrecognized molecular mechanism, while recognizing that future chemical optimization or formulation strategies (e.g., nanocarriers or structural analogs) are required to translate this mechanism into therapeutic benefit.

## 2 Materials and methods

### 2.1 Study design

TGF-β1-stimulated cells and bleomycin (BLM)-induced mice are the well-accepted cell and animal models of pulmonary fibrosis. Rutin (purity 98.09%), extracted from licorice, was purchased from MedChemExpress (MCE). Pirfenidone was purchased from Beijing Contini Pharmaceutical Co., Ltd. Nintedanib was purchased from Meilun Biotechnology Co., Ltd. Pirfenidone and nintedanib were used as positive controls to validate the responsiveness of cellular and animal models to well-established anti-fibrotic agents. This study was not designed for formal head-to-head comparison of efficacy or potency between rutin and these two drugs. The experimental design was shown in Figure 1.

**FIGURE 1:**
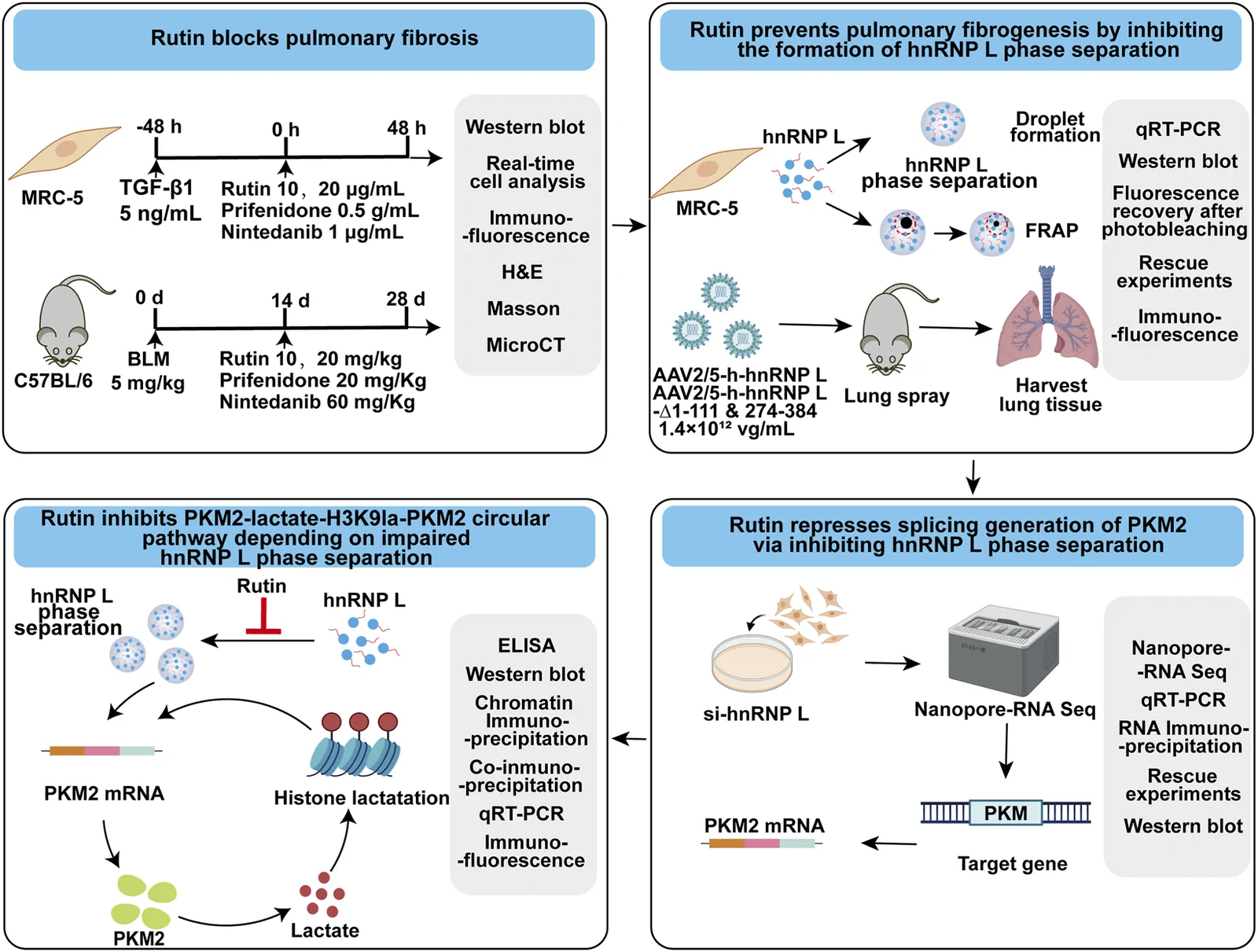
Experimental designed flowchart.

### 2.2 Cell culture

Human fetal lung fibroblast MRC-5 cells (Li X. et al., 2024) were purchased from the American Type Culture Collection (CCL-171) and cultured in minimum essential medium (MEM) containing 10% fetal bovine serum, 1% sodium pyruvate, 1% non-essential amino acids (NEAA), 1% GlutaMAX and 100 × penicillin/streptomycin solutions in a 37 °C and 5% CO_2_ incubator. Human fetal lung fibroblast IMR-90 cells (ATCC CCL-186) were cultured in MEM containing 10% fetal bovine serum and 1% penicillin/streptomycin at 37 °C with 5% CO_2_. Cells were first treated with 5 ng/mL TGF-β1 for 72 h, then treated with different concentrations of rutin for 48 h or transfected with plasmid of EGFP-hnRNP L according to different experiments. Lactic acid used in Supplementary Figure S1 experiments was purchased from MCE (catalog No. HY-B222), dissolved in cell culture medium, and administered to cells at 10 mM for 24 h. PKM-IN-1 employed in subsequent experiments was obtained from MCE (catalog No. HY-103617), dissolved in cell culture medium, and applied to cells at 2 μM for 12 h.

### 2.3 Animal model and ethical statement

Animal experiments were performed in accordance with the regulations of the Animal Experiment Ethics Committee of Shandong Medical and Pharmaceutical University. The ethical approval number for the animal experiments was 2022-175 and was obtained on 22 November 2022. Eight-week-old C57BL/6 mice (20 ± 5 g) were randomly grouped according to the random number table. There were 10 mice in each group. The BLM group was sprayed with 5 mg/kg of BLM (Nippon Kayaku Co., Ltd.) and the sham group was sprayed with the same amount of normal saline using the Micro Sprayer Aerosolizer (Shanghai Yuyan Scientific Instrument Co., Ltd.). Rutin was suspended in sterile saline containing 0.5% sodium carboxymethyl cellulose (CMC-Na) to a final concentration of 2 mg/mL. The suspension was prepared fresh daily, vortexed vigorously for 1 min before each gavage, and administered orally at a dose of 5 or 10 mL/kg body weight (equivalent to 10 or 20 mg/kg). Pirfenidone and nintedanib were prepared in the same manner according to the manufacturers’ instructions and administered at the indicated doses. Adeno-associated viruses (AAV2/5) -h-hnRNP L NC, AAV2/5-h-hnRNP L and AAV2/5-h-hnRNP L-Δ1-111 and 274–384 were purchased from He Yuan Biotechnology Co., Ltd. and sprayed into the mice’s lungs. The study protocol pre-specified n = 6 per group for all primary analyses. Ten mice per group were initially included to allow for potential attrition (e.g., mortality during BLM instillation or gavage, or failure of AAV transduction) and to ensure that at least six independent biological replicates would be available for statistical analysis. In the present study, no mortality or unexpected events occurred in any group; therefore, all ten mice per group completed the experiment. According to the pre-specified protocol, six mice per group were randomly selected from the ten mice using a computer-generated random number sequence (SPSS 26.0) by an observer blinded to group allocation. These six mice served as biological replicates for all primary endpoints. The remaining four mice per group were euthanized, and their lung tissues were preserved for future exploratory analyses not included in this manuscript. The right lung lobes of the six selected mice were harvested and immediately frozen in liquid nitrogen, which were subsequently utilized for the extraction of RNA and protein. The left lung lobes of the six selected mice were prepared for histological analyses to evaluate structural and fibrotic changes.

### 2.4 Molecular docking of rutin with hnRNP L

The hnRNP L crystal structure and rutin 3D structure were obtained from AlphaFold and the PUBCHEM Data Bank (Khirallah et al., 2022), respectively. Molecular docking was performed using AutoDock Vina 1.2.3 software. First, water, ions, and small molecules were removed from the hnRNP L protein via PyMol 2.5.5 software, then rutin and hnRNP L were converted to PDBQT format using ADFRsuite 1.03. Finally, the docking results were visually analyzed by PyMol 2.5.5.

### 2.5 Enhanced green fluorescent protein (EGFP)-hnRNP L protein purification experiment

The EGFP-hnRNP L gene was cloned into the pET28a (Kan+) expression vector and transformed into BL21 (DE3). Positive clones expressing the target protein were fermented in Luria-Bertani medium. Cells were collected by centrifugation and resuspended in 20 mM Tris, 450 mM NaCl, pH 8.0. The resuspended cell pellet was lysed by sonicating on ice. Cell supernatant was collected, filtered through a 0.22 μM membrane, and loaded onto a Ni-NTA column, and eluted with 20 mM Tris, 450 mM NaCl, 250 mM imidazole, pH 8.0. The eluent fractions were loaded onto a Superdex200 column (Cytiva), and eluted with 20 mM Tris, 450 mM NaCl, pH 8.0. Finally, EGFP-hnRNP L protein was purified. The purity of the protein was analyzed and quantified by using sodium dodecyl sulfate-polyacrylamide gel electrophoresis (SDS-PAGE). The protein concentration was measured by using NANODROP, and the extinction coefficient was 0.822.

### 2.6 Droplet formation assay

A solution of purified EGFP-hnRNP L and EGFP protein was mixed with 10% polyethylene glycol 6000 (PEG6000) and thoroughly combined. The mixture was poured into a 1.5 cm glass dish to observe droplet structure formation using a laser scanning confocal microscope (Leica, Germany).

### 2.7 Fluorescence recovery after photobleaching (FRAP)

MRC-5 cells were inoculated into a glass bottom specific for laser scanning confocal microscopy. When the cell density reached up to 65%, the plasmids of EGFP-hnRNP L were transfected into MRC-5 cells. The plasmids used were all purchased from Shandong Research Cloud Biotechnology Co., Ltd. Subsequently, the cells were washed with 1 × PBS for twice. Then, 5 μL of purified EGFP-hnRNP L was added into the glass dish. The punctate nuclei of single cells and the single droplet structure of purified protein were photobleached using 100% excitation light for 10 s and allowed to recover for about 2 min under the laser scanning microscope.

### 2.8 ChIP-PCR analysis

ChIP-PCR was performed by using the SimpleChIP® enzymatic chromatin IP kit (Cell Signaling Technology, United States) according to the manufacturer’s instructions. The cell samples were collected and incubated with formaldehyde for 10 min 2.5 M glycine was added and mixed thoroughly. After centrifugation, the precipitation was prepared for nuclear preparation and chromatin digestion. The chromatin was immunoprecipitated with anti-H3K9la or rabbit IgG antibodies and incubated overnight. Thirty microliters of ChIP-grade protein G agarose beads were added and incubated for 2 h. NaCl and protease K were added to the mixture, and the enriched DNA-protein complex was de-crosslinked to release DNA fragments. DNA was purified using a DNA centrifuge column and amplified by PCR.

### 2.9 Nanopore RNA sequencing

Total RNA from MRC-5 cells grouped into TGF-β1 + si-hnRNP L NC and TGF-β1 + si-hnRNP L was extracted by using the Total RNA Kit. 500 ng RNA was taken and the volume adjusted the volume to 9 μL with Nuclease-Free Water. The cDNA-PCR Sequencing Kit from Oxford Nanopore (SQK-PCS109) was used to prepare full-length cDNA libraries. Then the cDNA was amplified by PCR for 13–14 cycles with specific barcoded adapters from the Oxford Nanopore PCR Barcoding Kit. Finally, the 1D sequencing adapter was ligated to the DNA before loading onto a FLOPRO002 R9.4.1 flow cell in a PromethION sequencer. MinKNOW was used to run the sequencing with 48–72 h.

### 2.10 Co-immunoprecipitation (Co-IP)

The Co-IP kit (Absin, Shanghai, China) was used according to the instructions of the kit. First, 1 × 106 cells were collected, treated with pre-cooled lysis bufer for 5 min, and ultrasonically crushed on ice 3 times. After centrifugation, the supernatant was treated with 5 μL ProteinA/G and incubated at 4 °C for 1 h. After centrifugation again, the 5 μg antibody of rabbit IgG (Cell Signal Technology) and 5 μg rabbit anti-P300 (Cell Signal Technology) were added to the supernatant and incubated at 4 °C overnight. The next day, the supernatant was mixed with 5 μL Protein A/G and incubated at 4 °C for 3 h. After centrifugation, the precipitate was washed 3 times with washing buffer, treated with 1 × SDS to collect protein samples, and then analyzed.

### 2.11 Cell counting kit-8 (CCK-8) experiment

A total of 5 × 103 MRC-5 cells were cultured in the 96-well plate for 24 h. Different concentrations of rutin, each at 100 μL, were added to each well and incubated for either 24 or 48 h. Then, 10 μL of CCK-8 solution was added to each well. The absorbance was measured at 450 nm with a microplate reader after 4 h. Cell viability was calculated by using the appropriate formula.

### 2.12 Hematoxylin and Eosin (H&E) staining and Masson’s trichrome staining

Lung tissues fixed in paraformaldehyde were dehydrated in a graded ethanol series (Mohamed et al., 2023), soaked in paraffin overnight, embedded using paraffin, and then cut into 5 μm sections with a Leica microtome (RM2255). Paraffin sections were deparaffinized, dehydrated with absolute ethanol, and stained with H&E or Masson’s trichrome staining kit (Solarbio, China). After staining, the sections were cleared in xylene, dropped with neutral gum and sealed with a cover slip. The lung tissues of the mice were observed under a microscope.

### 2.13 Flow-volume curve (FVC) testing

After intraperitoneal anesthesia, a transverse incision was made in the trachea of the mice, and a metal cannula was inserted into the incision. The plastic catheter was attached to the pulmonary function instrument (DSI Buxco, United States) and mechanical ventilation was performed at a respiratory rate of 150 breaths/min, a tidal volume of 10 mL/kg, and a positive end-expiratory pressure of 3 cm H2O. The FVC data was obtained from the instrument.

### 2.14 Western blot

The treated cells or lung tissues were collected and lysed in RIPA lysis buffer (1:100) containing phenylmethylsulfonyl fluoride, and the corresponding protein concentrations were determined using a bicinchoninic acid protein assay kit (Coolaber, China). Proteins were separated by sodium dodecyl sulfate-polyacrylamide gel electrophoresis and then transferred to polyvinylidene fluoride membrane (El Behery et al., 2025). The protein bands were blocked with 5% nonfat dry milk for 2 h, and incubated with anti-collagen I (1:1,000, Affinity, China), anti-α-SMA (1:1,000, Affinity, China), anti-Vimentin (1:1,000, Affinity, China), Anti-GAPDH (1: 10,000, Affinity, China), anti-FAP1 (1:1,000, Cell Signaling, United States), anti-hnRNP L (1:1,000, Cell Signaling, United States),and anti-H3K9la (1:800, PTM Bio, China) and incubated overnight. The bands were washed using a Tris (hydroxymethyl) aminomethane buffer salt solution containing Tween 20 (TBST), then incubated with goat anti-rabbit/mouse secondary antibody for 1 h. Finally, the expression of protein was detected by extremely high sensitivity chemiluminescence kit (Spark Jade, China).

### 2.15 Quantitative real-time polymerase chain reaction (qRT-PCR)

Total RNA was extracted from cells or tissues (Alzamami et al., 2023) by using the AG RNAex Pro reagent and complementary DNA (cDNA) synthesis was performed by using the Evo M-MLV RT Premix reagent (Accurate Biology, AG11706) according to the manufacturer’s instructions. qRT-PCR was performed on the LightCycler 96 real-time PCR system by using 2 × SYBR Green Pro Taq HS Premix (Accurate Biology, A3A2291). A total volume of 20 μL system was as follows: 2 μL cDNA, 7.2 μL RNA-free water, 0.4 μL hnRNP L forward primer, 0.4 μL hnRNP L reverse primer, 10 μL SYBR® Premix Ex Taq™. The reaction program was as follows: initial denaturation at 95 °C for 5 s, followed by 40 cycles of PCR amplification at 95 °C for 10 s, 60 °C for 30 s and 72 °C for 30 s. Melting curve analysis at 95 °C for 10 s, 60 °C for 60 s and 95 °C for 1 s. Cooling at 37 °C for 1 s. The following primers were used: GAPDH, Forward Primer 5′ to 3′, GCA​CCG​TCA​AGG​CTG​AGA​AC, Reverse Primer 5′ to 3′, TGG​TGA​AGA​CGC​CAG​TGG​A; hnRNP L, Forward Primer 5′ to 3′, GAG​TTC​AGG​CGA​TGG​TGG​AA, Reverse Primer 5′ to 3′, AGC​GTG​TAG​GCT​TTG​CGT​AT; PKM, Forward Primer 5′ to 3′, ATT​ACA​GAC​GTG​CAC​CAC​CA, Reverse Primer 5′ to 3′, CGC​CTG​TAA​TCC​CAA​CAC​TT; PKM1, Forward Primer 5′ to 3′, AGG​CAG​AGG​CTG​CCA​TCT​AC, Reverse Primer 5′ to 3′, TGC​CAG​ACT​TGG​TGA​GGA​CG; PKM2, Forward Primer 5′ to 3′, CTG​AGG​CAG​CCA​TGT​TCC​AC, Reverse Primer 5′ to 3′, TGC​CAG​ACT​CCG​TCA​GAA​CT.

### 2.16 Real-time cellular proliferation and migration analysis (RTCA)

A total of 5 × 10^4^ cells/mL cells were seeded in an E-plate for proliferation analysis and a CIM plate for migration analysis, respectively. The CIM plate contains an upper chamber and a lower chamber. 40 μL serum-free medium was added into the upper chamber. 160 μL serum medium was added into the lower chamber. The parameters of the RTCA DPlus instrument (ACEA Biosciences, Inc., Hangzhou, China) were set up. Then cell samples were placed in the instrument, which can automatically record real-time cellular proliferation or migration curves. Cell index representing the amount of proliferation/migration cells was calculated with RTCA software from ACEA Biosciences (San Diego, CA).

### 2.17 Wound healing assay

MRC-5 cells were cultured in 96-well plate. When the cell density reached 60%, the complete medium was replaced with serum-free medium with or without TGF-β1 for 72 h. After a scratch was made on the cell surface using a specific instrument, cells were washed twice with PBS to wash away the dead cells. Then, different concentrations of rutin were added to the plates and the plates were incubated in an IncuCyte S3 live-cell analysis system (Essen BioScience). The instrument can automatically detect and take pictures over time.

### 2.18 Immunofluorescence observation

Cell slides were placed in 24-well plates and an appropriate number of MRC-5 cells were inoculated. Each well was fixed with 4% paraformaldehyde for 30 min, permeabilized with 0.25% Triton, and then blocked with goat serum for 1.5 h, and incubated with anti-hnRNP L at 4 °C overnight. Fluorescently labeled secondary antibody was added, incubated at room temperature for 40 min in the dark, and then discarded. 200 μL DAPI (4′, 6-diamidino-2-phenylindole) was added to each well to stain cell nuclei for 5 min, during which time PBS was used to wash between each step. Finally, antifade mounting medium was dropped on the cell slides. All images were collected under a laser scanning confocal microscope (LEICA Stellaris 5, Germany).

### 2.19 Surface plasmon resonance (SPR)

The amine coupling method was used for protein immobilization. A CM5 (BR100530, Cytiva) Sensor Chip was activated with 1-ethyl-3 -(3-dimethylaminopropyl) carbodiimide (EDC, GE Healthcare) and N-hydroxysuccinimide (NHS, GE Healthcare) at a flow rate of 10 μL/min for 800 s. The ligand (20 μg/mL Recombinant Human hnRNP L (P8197, FineTest) in pH 4.5 sodium acetate (BR100350, Cytiva) buffer) was immobilized on channel 3 at 10 μL/min for 600 s, and channels were blocked with ethanolamine for 800 s. A reference channel (channel 1) used protein-free buffer. For interaction testing, 5% dimethyl sulfoxide (DMSO) (D8418, Sigma) in 1 × PBS-P+ (28995084, Cytiva) was used as running buffer. Solvent correction was done with 4%–6% DMSO mixtures. Rutin (HY-N0148, MCE) at concentrations (4,000, 2,000, 1,000, 500, 250, 125 and 0 μM) flowed at 30 μL/min for 60 s binding and 60 s dissociation. Data were collected, reference-subtracted, and globally fitted to a 1:1 Langmuir model to obtain a KD.

### 2.20 Micro-computed tomography (MicroCT) measurement

The small animal MicroCT imaging system (PerkinElmer, MA, United States) was warmed up with parameters pre-adjusted. Following intraperitoneal anesthesia, mice were positioned flat on the MicroCT platform. X-ray parameters were set to 90 kV and 88 μA. The computed tomography images were acquired with a field of view of 36 mm and an exposure time of 4 min. Two-dimensional tomographic images were obtained using the system’s imaging software. Finally, MicroCT images were generated and exported.

### 2.21 Cellular thermal shift assay (CETSA)

MRC-5 cells were stimulated with 5 ng/mL TGF-β1 for 72 h, followed by treatment with 20 μg/mL rutin or vehicle control for 48 h. Cells were harvested, washed with PBS, and lysed in RIPA buffer supplemented with protease inhibitors. Cell lysates were equally aliquoted and heated individually at 45, 50, 55, 60, 65, and 70 °C for 3 min using a thermal cycler, then cooled at room temperature for 3 min. The heated lysates were centrifuged at 20,000 × g for 20 min at 4 °C, and the soluble supernatants were collected. The levels of hnRNP L protein were detected by Western blot.

### 2.22 Intracellular lactate measurement (ELISA)

According to the manufacturer’s instructions, the intracellular lactate level was quantified using a lactate detection kit based on colorimetric ELISA (Sigma-Aldrich (Shanghai) Trading Co., Ltd.; product number: MAK329). In brief, cells were collected, washed, and lysed with lysis buffer. The lysis buffer was centrifuged at 12,000 × g for 10 min at 4 °C, and the supernatant was used for lactate determination. A standard curve was drawn using the lactate standards provided in the kit, with lactate concentrations expressed in micrograms per liter (μg/L). The lactate content in the test samples was calculated by substituting the absorbance value into the standard curve and was reported as micrograms per liter (μg/L).

### 2.23 Statistical analysis

All quantitative data are presented as mean ± standard deviation (SD). The Shapiro–Wilk test and Levene’s test were first applied to assess normality and homogeneity of variances, respectively. For normally distributed data with equal variances, pairwise comparisons were made using the unpaired two-tailed Student’s *t*-test, while multiple-group comparisons were made with one-way ANOVA followed by Tukey’s HSD *post hoc* test. For each animal, three technical replicates were performed per assay, averaged to yield a single value per mouse, and only these averaged values were used as independent biological observations. *In vitro* data were derived from three independent experiments, each performed in technical triplicate to ensure reproducibility. All statistical analyses were performed in GraphPad Prism using Student’s t-test for pairwise and ANOVA for multiple comparisons. Statistical significance was indicated as follows: *p < 0.05; **p < 0.01.

## 3 Results

### 3.1 Rutin has the anti-pulmonary fibrotic effect *in vitro*

First, the rutin’s toxicity was assessed by a CCK-8 assay in normal MRC-5 cells. The cells were incubated in media containing varying concentrations of rutin for 24 and 48 h. The half-maximal inhibitory concentration (IC_50_) values for rutin were approximately 250 μg/mL at 24 h and 200 μg/mL at 48 h (Figure 2A). Then, fibroblast-to-myofibroblast differentiation model was established by using TGF-β1 stimulation. The results illustrated that rutin inhibited the growth of TGF-β1-treated cells in a time- and concentration-dependent manner. The IC_50_ values of rutin for TGF-β1-activated cells were approximately 60 μg/mL at 24 h and 40 μg/mL at 48 h. Notably, the IC_50_ values for rutin in TGF-β1-activated cells (60 μg/mL at 24 h) were significantly lower than in normal cells (250 μg/mL), indicating a selective sensitivity of the pathological phenotype. These findings suggest that rutin selectively inhibits the growth of cells treated with TGF-β1. Since rutin exerted a significant inhibitory effect starting from 10 μg/mL (Figure 2B), concentrations of 10 and 20 μg/mL rutin were selected for further studies. Meanwhile, pirfenidone and nintedanib were used as the positive control drugs to evaluate the anti-fibrotic effect of rutin. According to the literature (Hisatomi et al., 2012; Rangarajan et al., 2016; Yang Y. et al., 2024), 0.5 mg/mL and 20 mg/kg pirfenidone, and 1 μg/mL and 60 mg/kg nintedanib were used for cell and animal experiments, respectively. The real-time cellular analysis system showed that rutin inhibited the proliferation and migration of activated fibroblasts (Figure 2C), and this finding was further confirmed by the IncuCyte S3 instrument (Figure 2D). Immunofluorescence experiments revealed that rutin reduced the expression levels of α-SMA, a specific marker for myofibroblast differentiation. Concomitantly, rutin caused the morphology of myofibroblasts to become more regular and rounded (Figure 2E). Western blot analysis showed that rutin downregulated the expression of fibroblast activation protein 1 (FAP1) and fibrotic markers including vimentin, collagen I, and α-SMA (Figure 2F). The positive control drugs nintedanib and pirfenidone also demonstrated the expected anti-fibrotic effect in this model. These findings indicated that rutin can inhibit the differentiation of fibroblasts to myofibroblasts, as well as their proliferation, migration, and extracellular matrix deposition, and suggested that rutin possesses anti-pulmonary fibrotic property.

**FIGURE 2:**
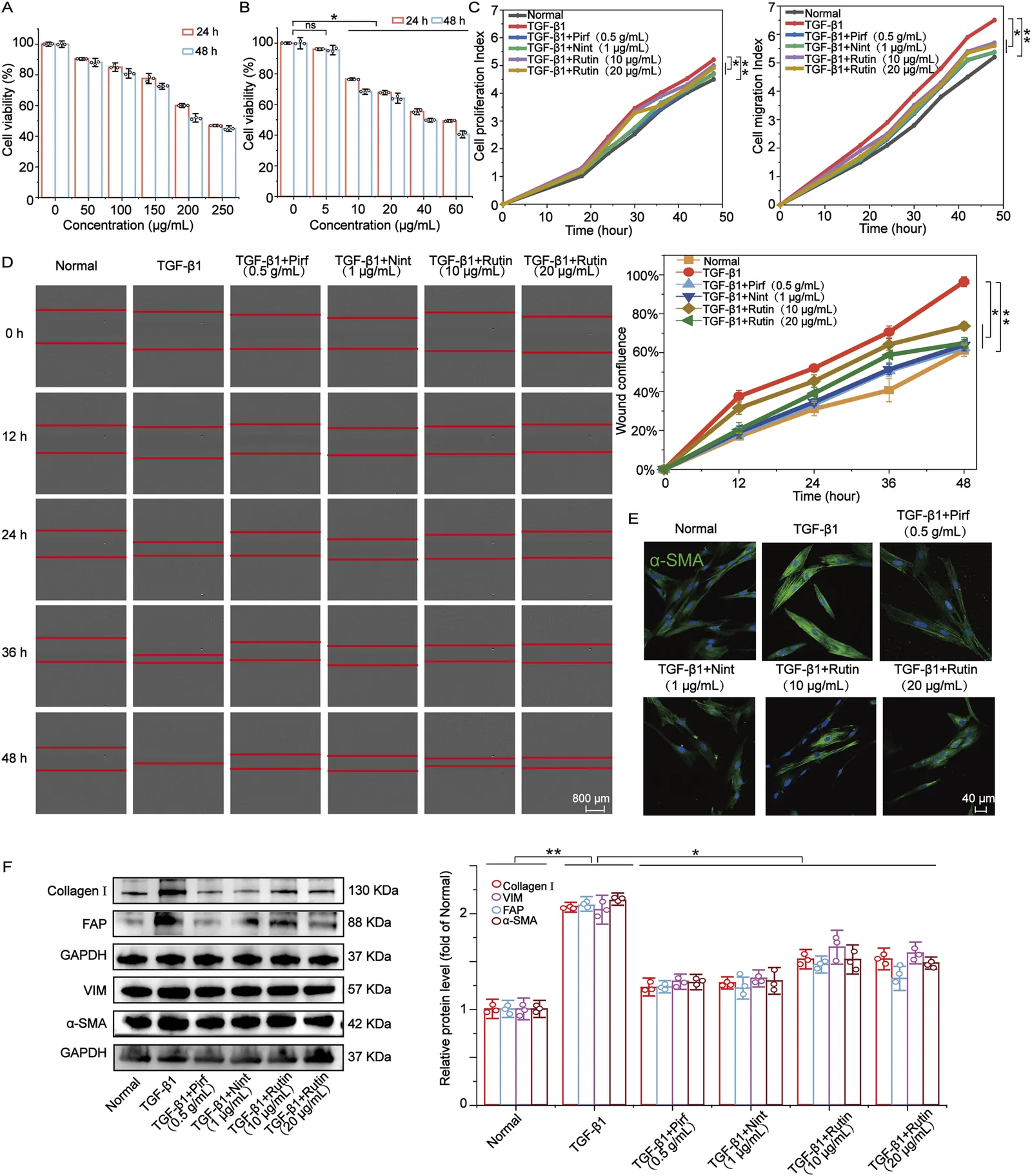
Screening of rutin concentration and its anti-pulmonary fibrotic effect in cells. **(A)** The CCK-8 assay was conducted to evaluate the cytotoxicity of rutin in normal cells. **(B)** Viability of TGF-β1-stimulated MRC-5 cells after rutin treatment. **(C)** The real-time cell analysis system shows the proliferation and migration of fibroblasts in each group. **(D)** The results of the migration assay were further validated using an IncuCyte S3 instrument. **(E)** Immunofluorescence staining for α-SMA (red). Nuclei were counterstained with DAPI (blue). **(F)** Western blot analysis and quantification of FAP1, vimentin, α-SMA, and collagen. **(I)** GAPDH was used as the loading control. Each bar represents the mean ± SD; n = 3, *p < 0.05, **p < 0.01.

### 3.2 Rutin attenuates pulmonary fibrosis *in vivo*

The anti-fibrotic effect of rutin was further evaluated in BLM-treated mice (Figure 3A). The flow-volume curve in the pulmonary function assessment demonstrated that rutin improved lung function in mice (Figure 3B). Dynamic lung compliance (Cdyn) was notably increased by rutin (Figure 3C). The total lung capacity of the rutin-treated group significantly exceeded that of the BLM group (Figure 3D). MicroCT imaging of small animals demonstrated prominent honeycomb lung and uneven patchy shadows in the BLM-treated group, whereas rutin treatment markedly alleviated these fibrotic features (Figure 3E). H&E and Masson trichrome images revealed that rutin diminished collagen deposition and the alveolar architecture appeared intact and clear compared with BLM treatment (Figure 3F). The expression levels of FAP1, vimentin, α-SMA, and collagen I were significantly downregulated by rutin (Figure 3G). All the above results also exhibited that 20 mg/kg rutin and the two active drugs could alleviate pulmonary fibrosis. Collectively, these findings indicate that rutin can attenuate pulmonary fibrosis. After establishing the antifibrotic effect of rutin *in vitro* and *in vivo*, we next sought to identify its direct molecular target. Previous work from our group has shown that hnRNP L is closely associated with the development of pulmonary fibrosis.

**FIGURE 3:**
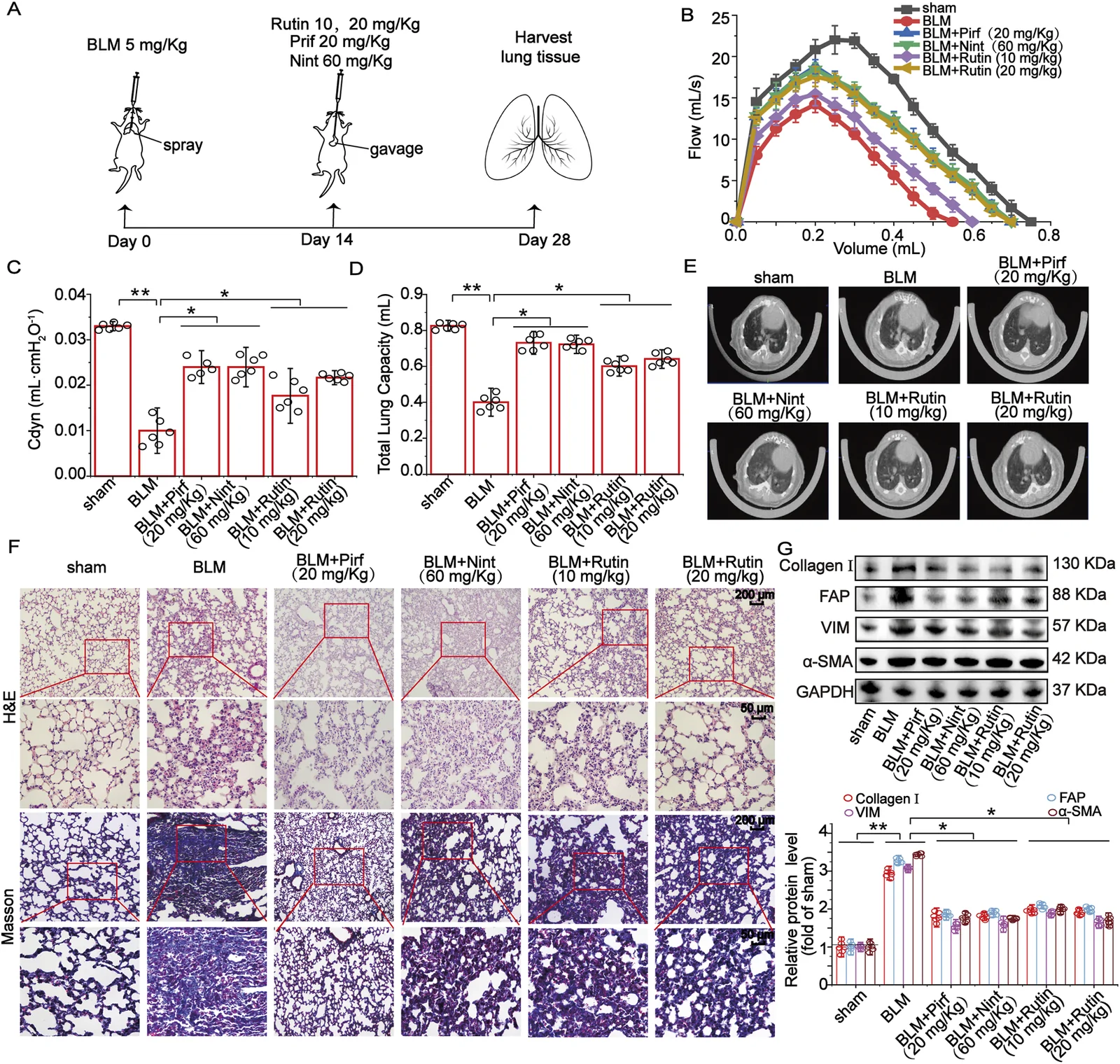
Rutin alleviated pulmonary fibrosis in mice treated with BLM. **(A)** The schematic representation depicts the administration of rutin in mice. **(B)** Representative flow-volume loops from pulmonary function tests. **(C)** Dynamic lung compliance (Cdyn). **(D)** Total lung capacity (TLC). **(E)** Micro-CT images of mouse lungs. **(F)** H&E and Masson trichrome staining of lung sections. **(G)** Western blot analysis of FAP1, vimentin, α-SMA, and collagen I in lung tissue. Each bar represents the mean ± SD; n = 6, *p < 0.05, **p < 0.01.

### 3.3 Rutin inhibits hnRNP L expression and attenuates pulmonary fibrogenesis

Next, we tested whether hnRNP L is a target of rutin. Molecular docking, performed with the AlphaFold-predicted structure revealed potential interactions with residues such as ASN-395, ARG-398, ASN-401, GLN-566, LYS-475, ASP-397, ASN-482, and PHE-561, as well as hydrophobic interactions involving PHE-477 and PHE-400, although this binding mode remains to be validated experimentally (Figure 4A). The surface plasmon resonance assay further confirmed that hnRNP L might be the target of rutin action. Purified hnRNP L proteins were immobilized on a CM5 chip with rutin flowing across their surface. The result showed that rutin bound to hnRNP L with equilibrium dissociation constant (K(D)) values of 19.48 μM (Figure 4B). To further verify whether rutin binds to hnRNP L in the cellular environment, we conducted a cellular thermal shift assay (CETSA). The results showed that in the rutin-treated group, the hnRNP L protein exhibited higher thermal stability across the tested temperature range compared to the TGF-β1 alone group (Figure 4C). Quantitative analysis revealed a significant rightward shift in the thermal denaturation curve after rutin treatment, indicating that rutin directly binds to hnRNP L in cells and stabilizes the protein against heat-induced denaturation. This evidence of intracellular binding corroborates the results from surface plasmon resonance and molecular docking, further strengthening the conclusion that hnRNP L is the direct target of rutin. qRT-PCR and Western blot analyses revealed that rutin inhibited hnRNP L expression at both the mRNA and protein levels compared with TGF-β1. As the inhibition by 20 μg/mL rutin was stronger than that by 10 μg/mL (Figures 4C,D), 20 μg/mL rutin was selected for the next cell experiments. An overexpressed plasmid for hnRNP L and an empty vector as a negative control were transfected into cells. Immunofluorescence experiments demonstrated that rutin reduced α-SMA expression compared to TGF-β1 treatment. Conversely, overexpression of hnRNP L reversed this inhibitory effect (Figure 4E). A scratch assay confirmed that hnRNP L overexpression counteracted the inhibitory effect of rutin on the migration of TGF-β1-treated cells (Figure 4F). Western blot rescue experiment also showed that hnRNP L overexpression reversed the downregulation of FAP1, α-SMA, vimentin, and collagen I induced by rutin (Figure 4G). These findings suggest that the anti-fibrotic effects of rutin are associated with the inhibition of hnRNP L expression. Next, we investigated the mechanism by which rutin regulates hnRNP L function. Given that many splicing factors exert their effects through liquid–liquid phase separation, we examined whether hnRNP L undergoes phase separation and whether this process can be modulated by rutin.

**FIGURE 4:**
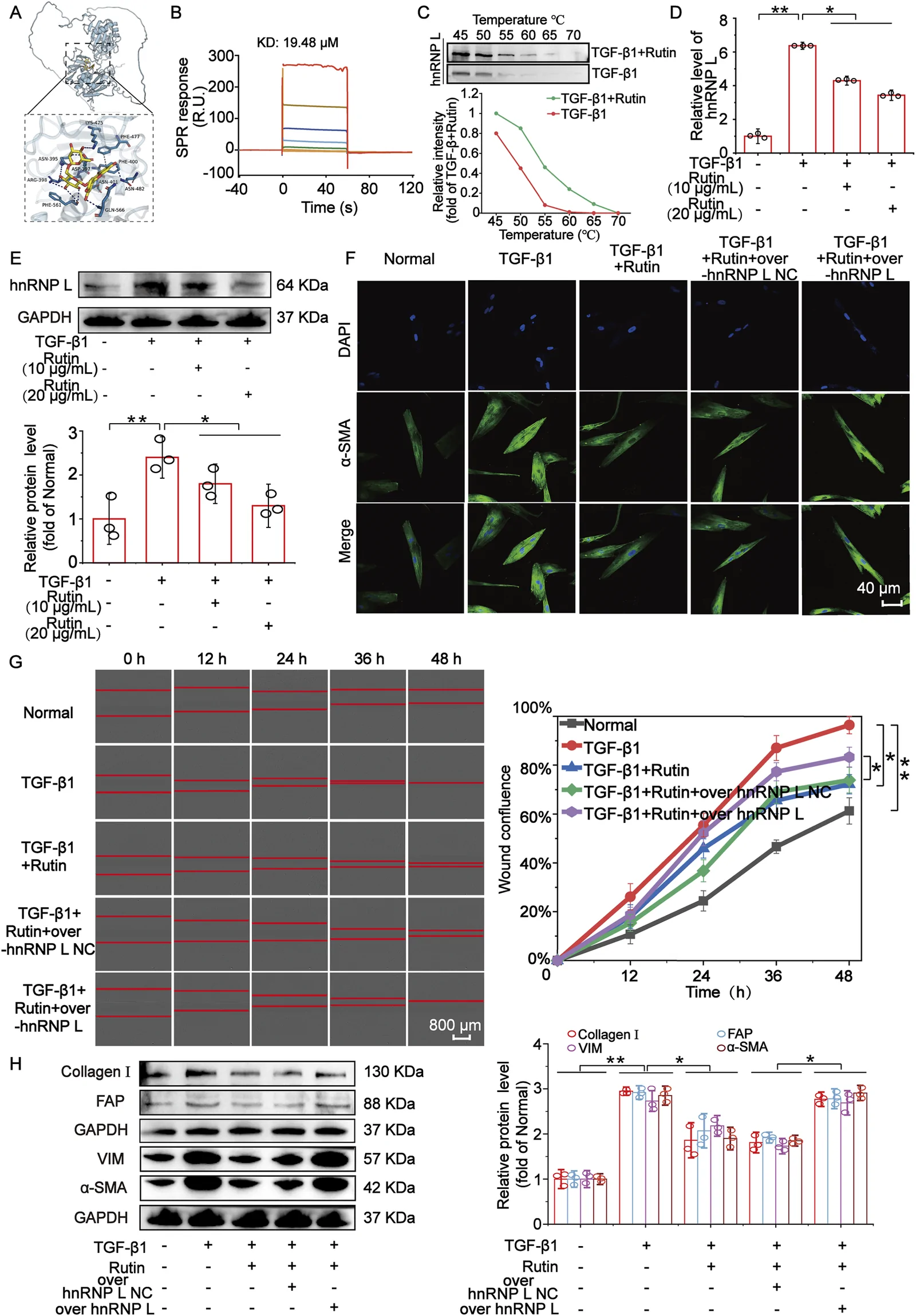
Rutin alleviated pulmonary fibrosis by weakening hnRNP L expression. **(A)** Molecular docking of rutin (yellow stick) with hnRNP L (light blue). Left: overview; right: local binding site. **(B)** Surface plasmon resonance (SPR) analysis of rutin binding to immobilized hnRNP L (KD ≈ 19.48 μM). **(C)** Rutin stabilizes hnRNP L protein in a cellular thermal shift assay (CETSA). **(D,E)** mRNA (qRT-PCR) and protein (Western blot) levels of hnRNP L after rutin treatment. **(F)** Immunofluorescence staining of α-SMA in cells transfected with empty vector or EGFP-hnRNP L, in the presence or absence of rutin. **(G)** Wound healing migration of the indicated groups assessed by IncuCyte. **(H)** Western blot analysis of FAP1, α-SMA, vimentin, and collagen I in hnRNP L rescue experiment. Each bar represents the mean ± SD; n = 3, *p < 0.05, **p < 0.01.

### 3.4 Rutin interferes with hnRNP L phase separation, which is associated with attenuated pulmonary fibrosis

Recent studies have highlighted that the function of numerous proteins depends on phase separation. Proteins that undergo phase separation typically contain intrinsically disordered regions (IDRs). According to the PONDR database, hnRNP L contains two IDRs, spanning amino acid segments 1–101 and 336–383 (Figure 5A), therefore, it is speculated that hnRNP L can form phase separation. Thus, this study further investigates the impact of rutin on the phase separation of hnRNP L. First, an enhanced green fluorescent protein (EGFP) -hnRNP L was constructed to track and observe whether hnRNP L can form phase separation. EGFP-hnRNP L proteins were purified and mixed with a 1 × PBS solution containing 10% PEG6000. FRAP experiment was performed to test the droplet diffusivity or mobility. Under a Leica confocal microscope, EGFP-hnRNP L was observed to form droplets. After photobleaching a single condensate with a high-intensity laser, fluorescence rapidly recovered within 4 min (Figure 5B), indicating that hnRNP L can form phase separation *in vitro*.

**FIGURE 5:**
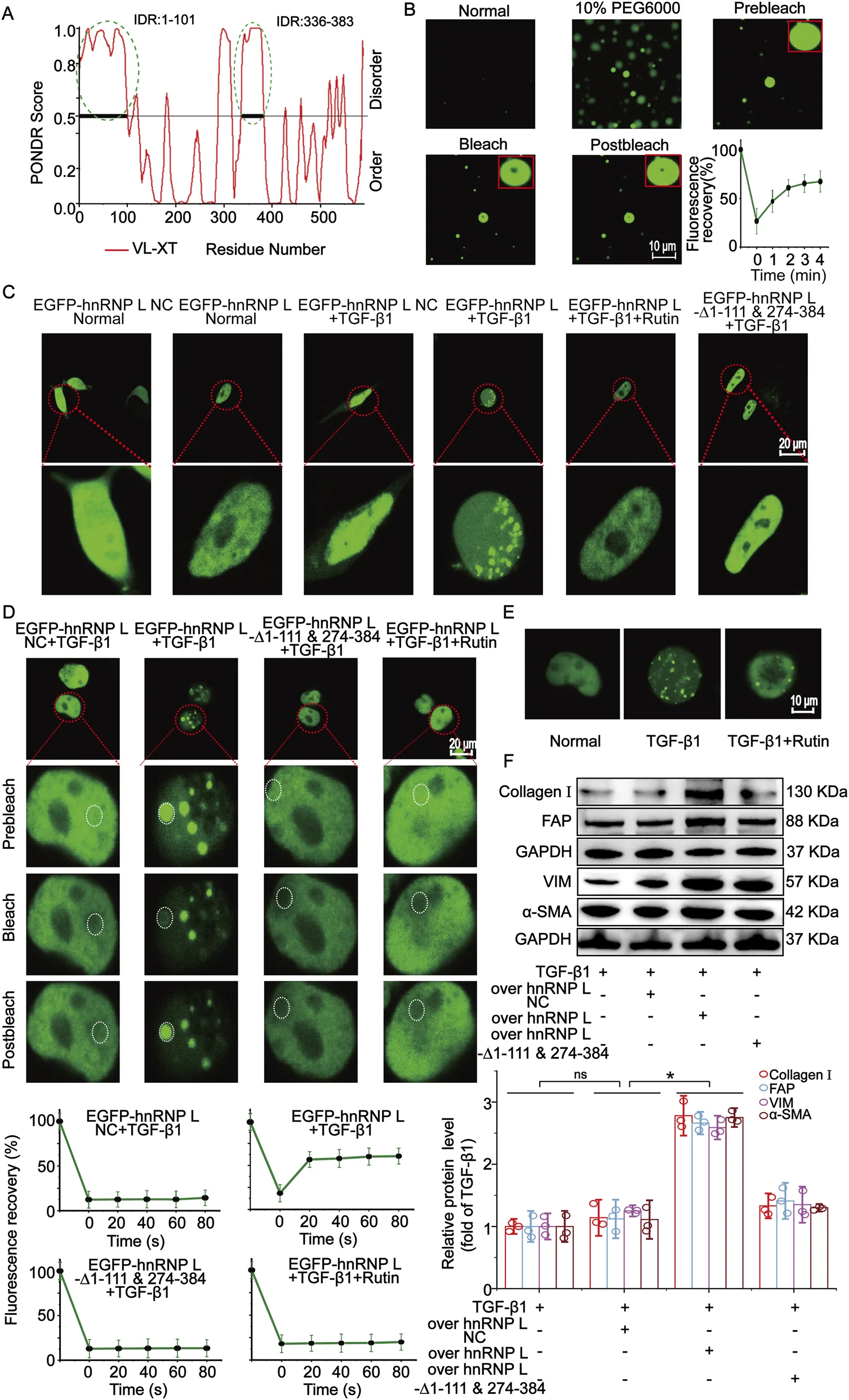
Rutin interferes with hnRNP L phase separation, which is associated with attenuated pulmonary fibrosis **(A)** PONDR database identifies the IDRs of hnRNP L. **(B)** Representative micrographs of EGFP-hnRNP L condensates before and after photobleaching were obtained using a laser scanning confocal microscope. **(C)** Confocal images of puncta for EGFP-hnRNP L or EGFP-hnRNP L Δ1–111/Δ274–384 in MRC-5 cells under the indicated treatments. **(D)** FRAP analysis was performed on nuclear puncta in the indicated groups. **(E)** The immunofluorescence experiment showed that rutin inhibited the formation of hnRNP L nuclear puncta. **(F)** Western blot analysis of fibrotic proteins in cells overexpressing hnRNP L or hnRNP L-Δ1-111 and 274-384. Each bar represents the mean ± SD; n = 3, *p < 0.05, **p < 0.01.

To further examine the phase separation role of hnRNP L during pulmonary fibrogenesis, plasmids encoding EGFP-hnRNP L (full-length hnRNP L) and EGFP-hnRNP L-Δ1-111 and 274-384 (truncated amino acid segments 1-111 and 274-384) were constructed. After transfection with EGFP-hnRNP L, TGF-β1 promoted hnRNP L phase separation formation and rutin prevented the formation of phase separation. When EGFP-hnRNP L-Δ1-111 and 274-384 was transfected, the phase separation did not form (Figure 5C). Upon photobleaching a single condensate in the EGFP-hnRNP L + TGF-β1 group, fluorescence recovery leveled off at 20 s. However, no fluorescence recovery was observed in the EGFP-hnRNP L-Δ1-111 and 274-384 group or rutin group (Figure 5D). These data confirmed that hnRNP L undergoes phase separation, which is dependent on the amino acid fragments 1-111 and 274-384, and rutin inhibits hnRNP L phase separation formation. Western blot analysis showed that hnRNP L overexpression aggravated the expression of pulmonary fibrotic proteins, whereas overexpression of hnRNP L-Δ1-111 and 274-384 had no significant impact on these proteins (Figure 5F). To further confirm the role of hnRNP L in the anti-fibrotic effect of rutin, we quantitatively measured α-SMA mRNA levels in IMR-90 cells (another human lung fibroblast cell line) using quantitative real-time PCR. Overexpression of hnRNP L reversed the rutin-induced reduction in α-SMA mRNA levels, whereas overexpression of hnRNP L-Δ1–111 and hnRNP L-Δ274–384 exerted no significant effect on this rutin-mediated suppression (Supplementary Figure S3). These data indicate that hnRNP L possesses the capacity to form phase-separated condensates, dependent on the amino acid fragments 1-111 and 274-384, and that rutin inhibits this process. Functionally, the phase separation capacity of hnRNP L is required for its pro-fibrotic effects. Next, we verified this conclusion in mice.

### 3.5 Rutin’s interference with hnRNP L phase separation is associated with its anti-fibrotic effects in mice

In the present *in vivo* rescue study, all groups were analyzed with six mice per group, as pre-specified in the study protocol. The hnRNP L and hnRNP L-Δ1-111 and 274-384 were packaged into an adeno-associated viral vector (AAV2/5) and administered via aerosol into the lungs of mice. Because Figure 3 demonstrated that 20 mg/kg rutin had a better therapeutic effect, this concentration was used in the mice rescue experiments (Figure 6A). qRT-PCR results demonstrated a significant increase in the expression levels of hnRNP L and hnRNP L-Δ1-111 and 274-384, indicating the adeno-associated viral vectors were transfected successfully (Figure 6B). The FVC in the pulmonary function assessment revealed that overexpression of hnRNP L deteriorated lung function, negating the beneficial effects of rutin. Conversely, overexpression of hnRNP L-Δ1-111 and 274-384 did not impair lung function and did not reverse rutin therapeutic effect (Figure 6C). Rutin significantly increased dynamic lung compliance (Cdyn) compared with the control group; however, hnRNP L overexpression reversed its therapeutic effects. In mice overexpressing hnRNP L-Δ1-111 and 274-384, lung compliance remained stable, and rutin treatment efficacy was preserved (Figure 6D). Total lung capacity (TLC) assessments indicated that rutin improved TLC. However, overexpressed hnRNP L led to a reduction in TLC, thereby negating the therapeutic benefit of rutin. Overexpressed hnRNP L-Δ1-111 and 274-384 did not affect TLC and preserved the rutin therapeutic effects (Figure 6E). MicroCT images revealed pronounced honeycomb-like lung structures and uneven patchy shadows in BLM-treated mice. Rutin treatment significantly alleviated these fibrotic characteristics, whereas hnRNP L overexpression reversed the positive effects of rutin. Overexpressed hnRNP L-Δ1-111 and 274-384 did not negate rutin therapeutic efficacy (Figure 6F). Histological analyses using H&E and Masson trichrome staining showed that rutin treatment effectively reduced pulmonary fibrosis. However, the hnRNP L overexpression group exhibited severe pulmonary fibrosis and increased collagen deposition, which hindered rutin anti-fibrotic effects. In the overexpressed hnRNP L-Δ1-111 and 274-384 group, the level of pulmonary fibrosis did not worsen, and rutin therapeutic effects remained intact (Figure 6G). Additionally, Western blot analysis confirmed that hnRNP L overexpression resulted in elevated levels of collagen, vimentin, α-SMA, and FAP1, which counteracted rutin’s inhibitory effects, while overexpression of hnRNP L-Δ1-111 and 274-384 did not counteract the beneficial effects of rutin (Figure 6H). Collectively, these findings support a model in which the anti-fibrotic effects of rutin are associated with interference of hnRNP L phase separation. Notably, these *in vivo* data were obtained from a single cohort of bleomycin-treated mice (n = 6 per group). Independent replication in additional animal cohorts will be important to further confirm the robustness of these mechanistic findings. We next investigated which downstream splicing events are regulated by hnRNP L phase separation.

**FIGURE 6:**
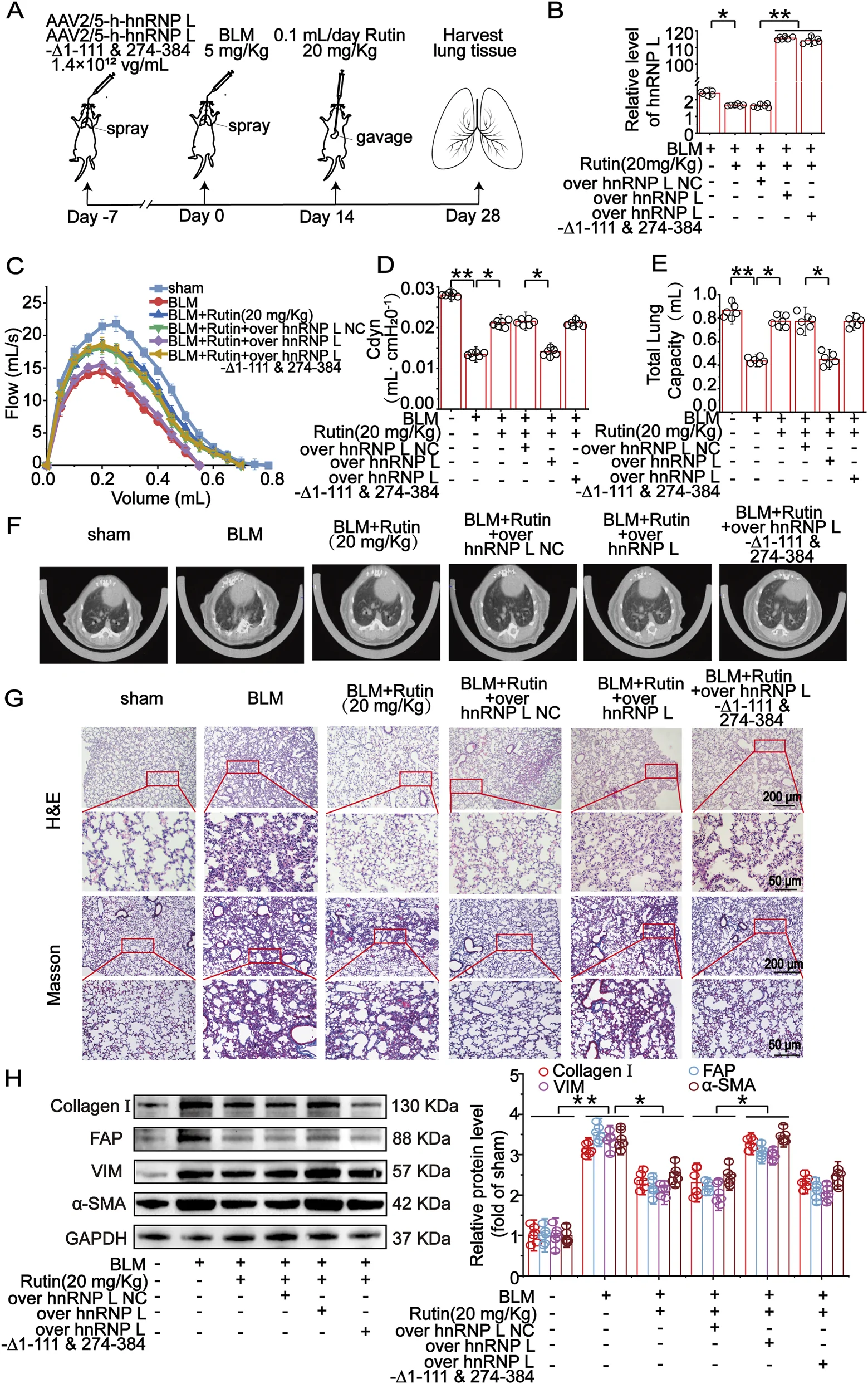
Rutin’s interference with hnRNP L phase separation is associated with its anti-fibrotic effects in mice. **(A)** A schematic representation of mice administered with BLM, rutin, overexpressed hnRNP L, and overexpressed hnRNP L-Δ1-111 and 274-384. **(B)** qRT-PCR validation of hnRNP L and hnRNP L-Δ1-111 and 274-384 overexpression in lung tissue. **(C)** Representative flow-volume loops from pulmonary function tests. **(D)** Dynamic lung compliance (Cdyn). **(E)** Total lung capacity (TLC). **(F)** Micro-CT images of mouse lungs. **(G)** H&E and Masson trichrome staining of lung sections. **(H)** Western blot analysis of FAP1, vimentin, α-SMA, and collagen I in lung tissue. Each bar represents the mean ± SD; n = 6, *p < 0.05, **p < 0.01.

### 3.6 Rutin modulates PKM2 alternative splicing in a manner associated with hnRNP L phase separation

Given that hnRNP L is a splicing factor, we conducted transcriptome sequencing on TGF-β1-stimulated MRC-5 cells after hnRNP L knockdown to explore its target genes and downstream pathways. Gene Ontology (GO) enrichment analysis indicated that the glycolytic process was one of the most significantly altered biological processes (Figure 7A, left). Kyoto Encyclopedia of Genes and Genomes (KEGG) pathway analysis further revealed that the differentially expressed genes were highly enriched in glycolysis (Figure 7A, right). Collectively, these results suggest that hnRNP L plays an important role in regulating glycolysis-related gene networks during pulmonary fibrosis. A heatmap generated by DESeq2 showed 21 differentially expressed genes associated with glycolysis in the TGF-β1 + si-hnRNP L group compared to the TGF-β1 group, with pyruvate kinase M (PKM) being one of the top-ranking genes. PKM expression was upregulated in the TGF-β1 group and downregulated in the TGF-β1 + si-hnRNP L group (Figure 7B). Using the Splicing Quantification and Differential Splicing Analysis 2 (SUPPA2) pipeline on our nanopore long read data, we identified 17,958 significant differential splicing events between the TGF β1 and si hnRNP L groups (Supplementary Figure S2A). Notably, 25 of these events were located in genes involved in glycolysis/gluconeogenesis (Supplementary Figure S2B). A heatmap of the top 50 differential splicing events (Supplementary Figure S2C) reveals that multiple glycolytic enzymes (e.g., PKM, PFKM, ALDOA, ENO1) exhibit hnRNP L dependent splicing changes, suggesting that hnRNP L phase separation may orchestrate a broader metabolic reprogramming beyond PKM alone. PKM can be spliced to produce PKM1 and PKM2, but the role of hnRNP L in PKM splicing during pulmonary fibrosis is still unknown. qRT-PCR results showed that rutin reduced PKM2 expression and increased PKM1 expression at the mRNA level, but overexpression of hnRNP L reversed the effects of rutin (Figure 7C). The RIP experiment elucidated that the PKM pre-mRNA bound to hnRNP L, and the binding amount decreased under rutin treatment compared with TGF-β1 treatment (Figure 7D). Western blot analysis confirmed that rutin significantly reduced PKM2 expression (Figure 7E), whereas hnRNP L interference led to downregulation of PKM2 (Figure 7F). In a rescue experiment, Western blot result showed that rutin reduced PKM2 expression, and overexpression of hnRNP L reversed this reduction (Figure 7G), but overexpression of hnRNP L-Δ1-111 and 274-384 did not reverse the increase in PKM1 and decrease in PKM2 in rutin-treated cells (Figure 7H). These findings suggest that rutin modulates PKM2 alternative splicing, and this modulation is associated with its interference of hnRNP L phase separation.

**FIGURE 7:**
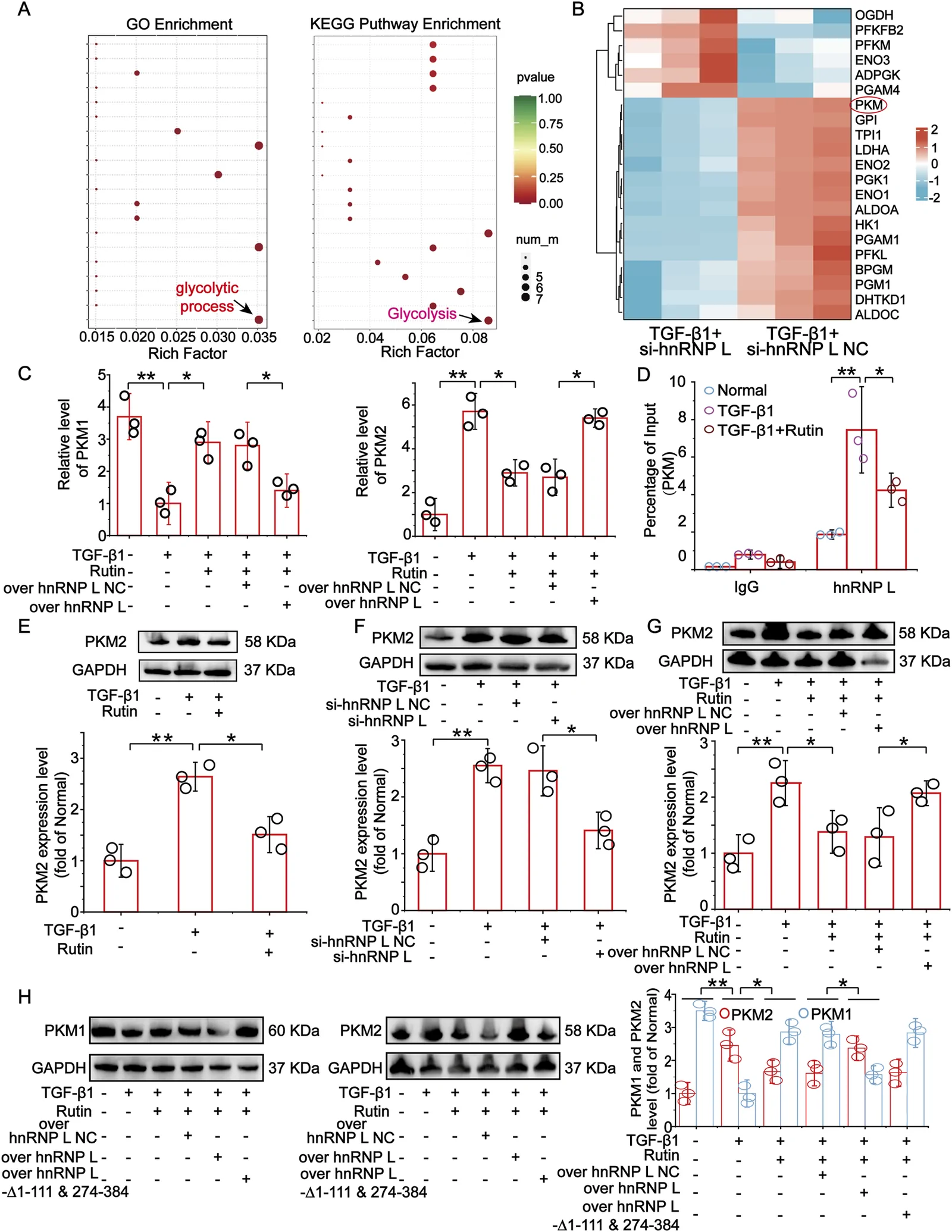
Rutin modulates PKM2 alternative splicing in a manner associated with hnRNP L phase separation. **(A)** GO biological process enrichment analysis (*left*) and KEGG pathway enrichment analysis (*right*) of differentially expressed genes between TGF-β1 + si-hnRNP L and TGF-β1 groups. **(B)** Heatmap showing 21 differentially expressed glycolysis-related genes (DESeq2). **(C)** qRT-PCR for PKM1 and PKM2 mRNA in the indicated groups. **(D)** RIP assay for hnRNP L binding to PKM pre-mRNA. **(E)** Western blot analysis of PKM2 protein after rutin treatment. **(F)** PKM2 protein levels after hnRNP L siRNA transfection. **(G)** PKM2 protein in rescue experiments with hnRNP L overexpression. **(H)** PKM1 and PKM2 protein levels in cells overexpressing hnRNP L variants. Each bar represents the mean ± SD; n = 3, *p < 0.05, **p < 0.01.

### 3.7 Rutin modulates the PKM2–lactate–H3K9la regulatory axis in a manner associated with hnRNP L phase separation

PKM2 is a key enzyme that produces lactate by enhancing glycolysis. ELISA-based quantification of intracellular lactate showed that lactate levels decreased after PKM2 inhibitor (PKM2-IN-1) intervention, and overexpression of PKM2 reversed this reduction (Figure 8A). Rutin and si-hnRNP L significantly reduced lactate levels, but hnRNP L overexpression raised lactate levels and reversed the inhibitory effects of rutin (Figure 8B), suggesting that the inhibition of lactate production by rutin depends on hnRNP L.

**FIGURE 8:**
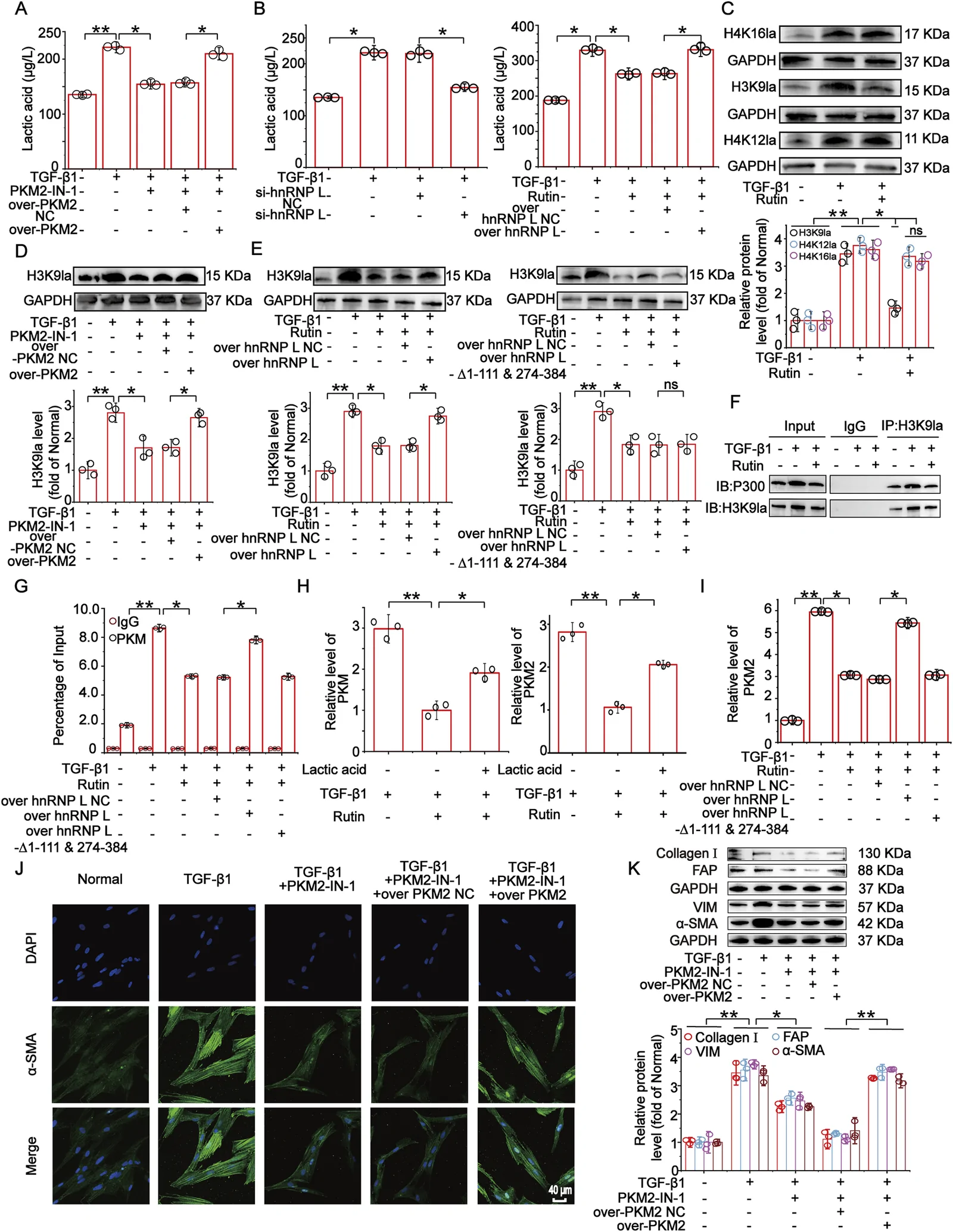
Rutin modulates the PKM2–lactate–H3K9la regulatory axis in a manner associated with hnRNP L phase separation. **(A)** Intracellular lactate levels (ELISA) after PKM2 inhibitor (PKM2-IN-1) and PKM2 overexpression. **(B)** Lactate levels in cells treated with rutin and hnRNP L overexpression. **(C)** Western blot analysis of histone lactylation marks (H3K9la, H4K12la, H4K16la). **(D)** H3K9la levels after PKM2-IN-1 treatment and PKM2 overexpression. **(E)** H3K9la levels in rescue experiments with hnRNP L and hnRNP L-Δ1-111 and 274-384. **(F)** Co-immunoprecipitation of P300 with H3K9la. **(G)** ChIP-PCR analysis of H3K9la enrichment at the PKM promoter. **(H)** qRT-PCR for PKM mRNA and PKM2 in TGF-β1-stimulated MRC-5 cells treated with rutin and sodium lactate. **(I)** qRT-PCR for PKM2 transcript in the same rescue groups as **(G)**. **(J)** Immunofluorescence staining for α-SMA after PKM2-IN-1 and PKM2 overexpression. **(K)** Western blot analysis of fibrotic proteins under the same conditions. Each bar represents the mean ± SD; n = 3, *p < 0.05, **p < 0.01.

Lactate can cause histone lactylation. However, the role of histone lactylation in pulmonary fibrosis remains unclear. Therefore, whether rutin influences histone lactylation was examined. First, we screened three lactylated histones—H3K9la, H4K12la, and H4K16la to determine which was affected by rutin. The results showed that rutin significantly reduced histone H3K9 lactylation (H3K9la) (Figure 8C), inhibition of PKM2 reduced H3K9la, and overexpression of PKM2 reversed this effect (Figure 8D). When TGF-β1-stimulated MRC-5 cells were treated with rutin in the presence of exogenous lactic acid, the inhibitory effects of rutin on H3K9la and the fibrotic marker α-SMA were significantly attenuated by the addition of lactic acid (Supplementary Figure S1). Co-IP experiment showed that P300, the enzyme responsible for lactylation modification, increased H3K9la in the TGF-β1 group, whereas rutin inhibited this effect (Figure 8F), indicating that the P300 enzyme was involved in the rutin inhibitory effect. In a rescue experiment, rutin reduced H3K9la expression, but overexpressed hnRNP L reversed this effect. Notably, overexpressed hnRNP L-Δ1-111 and 274-384 did not reverse the decrease of H3K9la in rutin-treated cells (Figure 8E). These results indicated that rutin reduces H3K9la by inhibiting hnRNP L-mediated PKM2 splicing generation.

H3K9la (protein) can regulate DNA transcription. Because ChIP-PCR can detect the interaction of protein and DNA, whether H3K9la regulates PKM2 transcription under rutin treatment was first explored by ChIP-PCR. Since PKM2 is transcribed from PKM DNA, primers for the PKM promoter were designed for ChIP-PCR to evaluate how H3K9la affects PKM transcription in response to rutin. The data demonstrated that TGF-β1 enhanced the interaction of H3K9la to the PKM promoter, whereas rutin decreased this interaction. Overexpression of hnRNP L reversed the effect of rutin, but hnRNP L-Δ1-111 and 274-384 had no this effect (Figure 8G), indicating that rutin can inhibit PKM transcription. To further determine whether lactate functionally drives PKM transcription through H3K9la, we performed a metabolite rescue experiment. Consistent with the reduced H3K9la occupancy detected by ChIP-PCR, rutin markedly decreased total PKM mRNA levels. Importantly, supplementation with exogenous lactic acid largely reversed this inhibitory effect and restored PKM pre-mRNA and PKM2 transcript abundance (Figure 8H). These data support a role for lactate in maintaining PKM transcription, consistent with a model in which the hnRNP L–PKM2–lactate axis contributes to sustaining PKM expression. Collectively, these data indicate that rutin treatment is associated with reduced PKM2 expression, decreased lactate production, and diminished H3K9la occupancy at the PKM promoter, and that overexpression of hnRNP L reverses these effects in a phase separation-dependent manner (Figure 8I).

Finally, immunofluorescence assay showed that PKM2 inhibitor (PKM2-IN-1) intervention inhibited the expression of pulmonary fibrosis-related proteins α-SMA and overexpression of PKM2 reversed this effect (Figure 8J). The rescue experiment of Western blot showed that the PKM2 inhibitor reduced the expression of fibrotic proteins. In contrast, overexpression of PKM2 countered the effects of PKM2-IN-1 and increased the expression of these proteins (Figure 8K). Collectively, these data indicate that rutin treatment is associated with reduced PKM2 expression, decreased lactate production, and diminished H3K9la occupancy at the PKM promoter, and that overexpression of hnRNP L reverses these effects in a phase separation-dependent manner.

## 4 Discussion

Currently, blocking fibroblast-to-myofibroblast differentiation is a key strategy for the development of anti-pulmonary fibrosis therapeutics (Luo et al., 2025; Peng et al., 2025). Rhizoma coptidis inhibits the transformation of lung fibroblasts into myofibroblasts (Yang J. et al., 2024). Atractylodinol significantly suppresses the fibroblast-to-myofibroblast transition to prevent pulmonary fibrosis *in vivo* and *in vitro* (Hao et al., 2023). The present study demonstrates that rutin inhibits fibroblast-to-myofibroblast differentiation and suppresses pulmonary fibrogenesis *in vitro* and *in vivo*. Mechanistically, our data support a model in which rutin may interfere with hnRNP L phase separation, and this interference is associated with modulation of the PKM2–lactate–H3K9la regulatory circuit (Figure 9).

**FIGURE 9:**
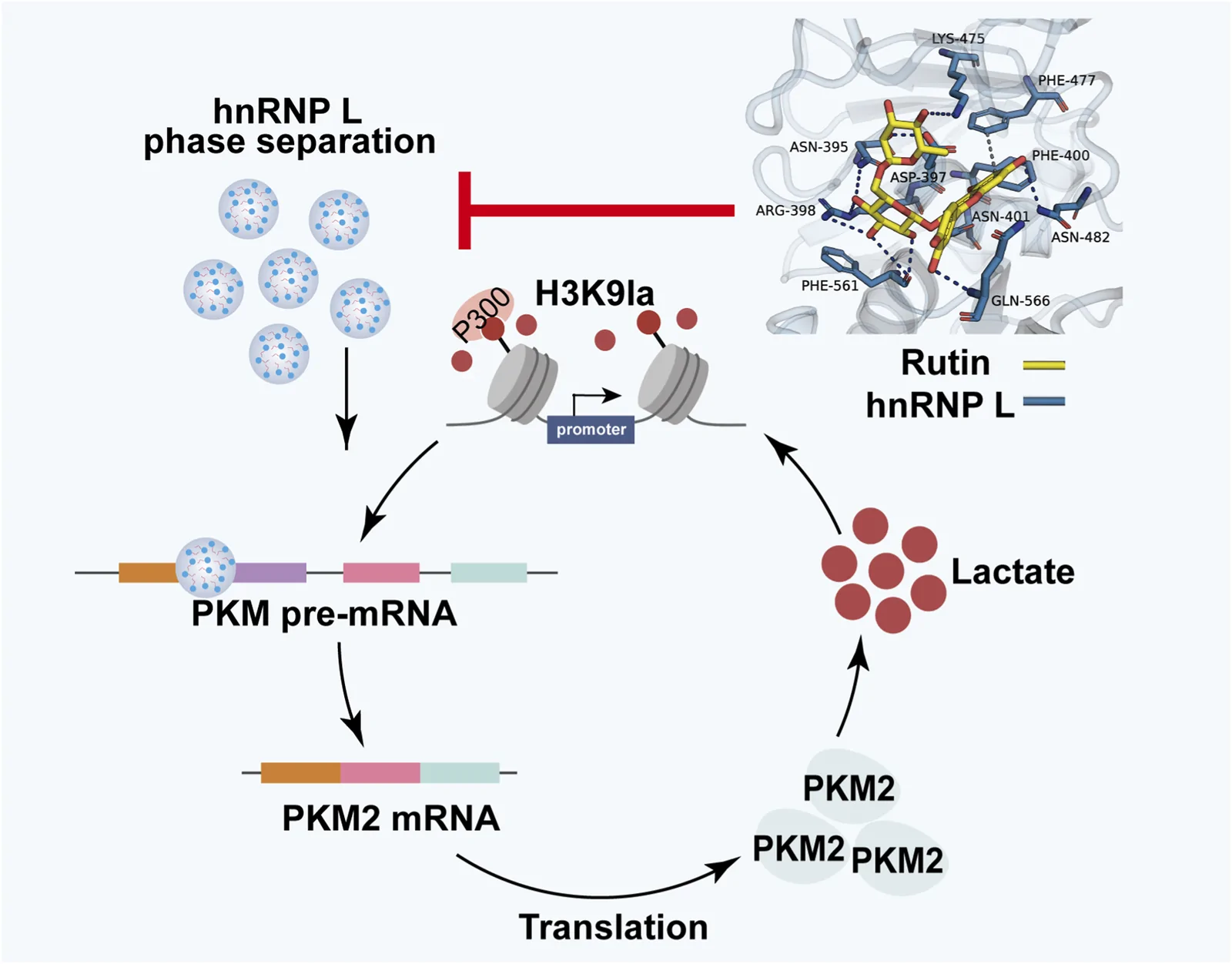
Rutin interferes with hnRNP L phase separation and modulates the PKM2–lactate–H3K9la axis in pulmonary fibrosis.

Recent studies have revealed that phase separation is associated with various diseases. For example, the phosphorylation-regulated dynamic phase separation of HIP-55 plays a protective role against heart failure (Jiang et al., 2024). During viral infection, AXIN1 undergoes phase separation induced by phosphorylated TANK-binding kinase 1, which enhances the antiviral immune responses of AXIN1 (Dai et al., 2024). Our group recently demonstrated that hnRNP L undergoes phase separation and that this capacity is required for its pro-fibrotic functions in pulmonary fibrosis (Wang M. et al., 2026). In the present study, we extend this finding by showing that rutin binds hnRNP L and interferes with its phase separation, and that this interference correlates with modulation of the PKM2–lactate–H3K9la axis. Our data show that rutin interferes with condensate assembly, and that this interference correlates with alterations in PKM splicing, downregulation of PKM2, and diminished lactate output. The ability of rutin to modulate phase separation provides new insights into drug–protein interactions.

PKM2, an isozyme of pyruvate kinase, is primarily involved in the final step of glycolysis. Its deletion significantly mitigates BLM-induced fibrosis progression by blocking myofibroblast differentiation (Gao et al., 2022). Shikonin, a PKM2 inhibitor, has been shown to restrain renal interstitial fibrosis and renal tubular apoptosis (Liu H. et al., 2024). Our study found that rutin treatment is associated with reduced lactate levels and decreased H3K9la, consistent with the notion that rutin may modulate this metabolic–epigenetic axis through its effects on PKM2-dependent glycolysis. This potential link has not been previously reported for rutin. Histone lactylation establishes an intrinsic connection between gene expression and cellular metabolism and is involved in numerous diseases (Qiu et al., 2025). In vascular smooth muscle cells, metabolic reprogramming mediated by tumour necrosis factor receptor-associated protein 1 can enhance lactate-dependent H4K12 lactylation via histone deacetylase 3, promoting the expression of the senescence-associated secretory phenotype (Li C. et al., 2024). In valve interstitial cells, lumican mediates H3 histonelactylation, which contributes to aortic valve calcification and leads to calcific aortic valve disease (Huang et al., 2024). Taken together, these results are consistent with a model in which rutin, by interfering with hnRNP L phase separation, modulates the PKM2–lactate–H3K9la regulatory axis to attenuate pulmonary fibrosis.

Previous studies have reported the anti-fibrotic effects of rutin in liver, kidney and lung models, mainly attributing its protection to the suppression of oxidative stress, inhibition of the TGF-β/Smad pathway, and anti-inflammatory activities (Karunarathne et al., 2024). However, these broad mechanistic explanations neither account for rutin’s selective action on pathological cells nor define a concrete druggable target. Our study provides a new conceptual framework by suggesting that rutin may interfere with the phase-separated splicing factor hnRNP L, which is associated with altered PKM2 alternative splicing and modulation of a metabolic–epigenetic regulatory circuit. This finding advances our understanding of how natural products can exert pharmacological functions through modulation of phase separation, a biophysical mechanism not previously linked to rutin’s action. Notably, rutin is a polyphenol with well-documented antioxidant and anti-inflammatory activities. The present study does not exclude the possibility that these properties contribute to its overall anti-fibrotic effects *in vivo*. The relative contribution of hnRNP L phase separation inhibition *versus* canonical Nrf2 activation or direct TGF-β receptor signaling modulation remains to be quantitatively dissected. It is plausible that rutin’s anti-fibrotic action results from a combination of effects on multiple targets; the model proposed here represents one facet of this polypharmacology.

Several limitations of the present study should be acknowledged. First, although CETSA and SPR support a direct interaction between rutin and hnRNP L, the binding affinity is moderate (KD ≈ 19 μM). It remains possible that rutin engages additional targets, and the anti-fibrotic effect observed may reflect cumulative modulation of multiple nodes rather than exclusive on-target activity. Second, the IDR-truncated hnRNP L mutant (Δ1–111 and 274–384) lost both phase separation ability and pro-fibrotic activity, but we cannot exclude the possibility that this truncation simultaneously impairs other functional domains of hnRNP L independent of phase separation. Definitive evidence that phase-separated hnRNP L condensates directly execute the PKM splicing switch will require *in vitro* reconstitution of condensates and splicing assays. Third, while we observed puncta suggestive of hnRNP L condensates in cells and *in vitro*, direct visualization of endogenous hnRNP L phase separation in fibrotic lung tissue has not been achieved; future studies using CRISPR-based endogenous tagging and validated phase separation-specific antibodies are needed. Fourth, our mechanistic studies were conducted in MRC-5 and IMR-90 fetal lung fibroblast lines, which may not fully recapitulate the behavior of primary adult lung fibroblasts or fibroblasts from IPF patients. Validation in disease-relevant primary cells is warranted. Furthermore, our models do not incorporate the mechanical microenvironment, particularly increased matrix stiffness, which is a principal regulator of PKM2 in lung fibroblasts. Future studies using stiffness-tunable substrates or decellularized fibrotic lung matrices will be needed to determine whether hnRNP L phase separation and the PKM2–lactate–H3K9la axis are also modulated by mechanical cues. Fifth, the hierarchical order of the PKM2–lactate–H3K9la circuit is inferred from pharmacologic and genetic perturbations combined with correlation analyses; however, the present data do not establish definitive causality at each step. Lactate may also derive from pathways other than PKM2-dependent glycolysis, and H3K9la may be regulated by additional histone modifiers beyond P300. Locus-specific manipulation of H3K9la at the PKM promoter will be necessary to establish a causal link between this epigenetic mark and PKM transcription. Six mice per group were randomly selected from the ten mice using a computer-generated random number sequence (SPSS 26.0) by an observer blinded to group allocation, which should reduce selection bias; however, the reduced effective sample size may have limited statistical power, and sampling variability cannot be completely excluded. The remaining four mice per group were not included in the primary endpoint analysis and were preserved for future exploratory analyses; therefore, a sensitivity analysis including all 10 mice per group could not be performed. In addition, all *in vivo* experiments, including the AAV-mediated rescue studies, were performed in a single cohort (n = 6 per group). Although randomized and blinded, and consistent with our *in vitro* findings, these results require independent replication in a separate animal cohort to fully confirm their robustness. Future studies will address this with additional cohorts and genetic models. Finally, the pharmacokinetic limitations of rutin, including its low oral bioavailability, restrict its immediate translational potential. In addition, we did not perform pharmacokinetic profiling of rutin or its metabolites in the present study. Future LC-MS/MS-based analyses of plasma, lung tissue, and urine will be necessary to correlate systemic exposure with target engagement and antifibrotic efficacy. In the future, we plan to adopt strategies such as nano-encapsulation, liposomal delivery, and structural derivatization to improve rutin’s bioavailability and therapeutic index. In the present study, rutin was therefore utilized as a tool compound to dissect a mechanistic framework; future chemical optimization or formulation strategies are required to translate these findings into therapeutic applications.

In summary, while several limitations remain to be addressed, the present study provides evidence supporting a previously unrecognized mechanistic framework—hnRNP L phase separation associated with PKM2 alternative splicing, lactate production, and H3K9la-mediated transcriptional regulation—and identifies rutin as a natural compound that may interfere with this regulatory interplay. This model provides a basis for future therapeutic targeting of phase separation in fibrotic diseases, though extensive validation in more physiological systems and optimization of rutin’s bioavailability are required before clinical translation can be considered.

## Data Availability

The original contributions presented in the study are included in the article; further inquiries can be directed to the corresponding authors. The Oxford Nanopore transcriptome sequencing data generated in this study have been deposited in the National Center for Biotechnology Information (NCBI) Sequence Read Archive (SRA) under the BioProject accession number PRJNA1491298.

## References

[B1] Alanazi I. M. Abo Nahas H. H. Mohamed D. I. Hosny N. Elewa Y. H. A. Agarwal M. (2025). Integrative metabolomic, network pharmacology, and experimental evidence for *Lepidium sativum* seed extract as a natural modulator of pulmonary fibrosis via the ncNRFR/Let-7d regulatory pathway. Pharmaceuticals 18, 1820. 10.3390/ph18121820 41471309 PMC13134761

[B2] Allen R. J. Oldham J. M. Jenkins D. A. Leavy O. C. Guillen-Guio B. Melbourne C. A. (2023). Longitudinal lung function and gas transfer in individuals with idiopathic pulmonary fibrosis: a genome-wide association study. Lancet. Respir. Med. 11, 65–73. 10.1016/S2213-2600(22)00251-X 35985358 PMC10077113

[B3] Alzahrani A. R. Mohamed D. I. Abo Nahas H. H. Alaa El-Din Aly El-Waseef D. Altamimi A. S. Youssef I. H. (2024). Trimetazidine alleviates bleomycin-induced pulmonary fibrosis by targeting the long noncoding RNA CBR3-AS1-mediated miRNA-29 and resistin-like molecule alpha 1: deciphering a novel trifecta role of lncRNA CBR3-AS1/miRNA-29/FIZZ1 axis in lung fibrosis. Drug. Des. Devel Ther. 18, 3959–3986. 10.2147/DDDT.S463626 39252766 PMC11382803

[B4] Alzamami A. Radwan E. M. Abo-Elabass E. Behery M. E. Alshwyeh H. A. Al-Olayan E. (2023). Novel 8-methoxycoumarin-3-carboxamides with potent anticancer activity against liver cancer via targeting caspase-3/7 and β-tubulin polymerization. BMC Chem. 17, 174. 10.1186/s13065-023-01063-5 38041156 PMC10693084

[B5] Chen S. Wang Y. Zhang J. Liu B. Liu W. Cao G. (2025). YTHDC1 phase separation drives the nuclear export of m^6^A-modified lncNONMMUT062668.2 through the transport complex SRSF3-ALYREF-XPO5 to aggravate pulmonary fibrosis. Cell. Death Dis. 16 (1), 279. 10.1038/s41419-025-07608-x 40221424 PMC11993731

[B6] Chianese M. Screm G. Salton F. Confalonieri P. Trotta L. Barbieri M. (2024). Pirfenidone and nintedanib in pulmonary fibrosis: lights and shadows. Pharm. (Basel) 17, 709. 10.3390/ph17060709 38931376 PMC11206515

[B7] Dai D. L. Xie C. Zhong L. Y. Liu S. X. Zhang L. L. Zhang H. (2024). AXIN1 boosts antiviral response through IRF3 stabilization and induced phase separation. Signal Transduct. Target Ther. 9, 281. 10.1038/s41392-024-01978-y 39384753 PMC11464762

[B8] El Behery M. Abo-Elmatty D. M. Alsunbul M. Mohey El-Deen Y. I. Mohamed D. I. Rashad Sindi E. (2025). Exploration of the anticancer efficacy of a novel 1,3-thiazole analog in an ehrlich ascites carcinoma model: *in vivo* and *in silico* insights into hepatorenal protective potentials via the modulation of apoptosis, oxidative stress and inflammation. RSC Adv. 15 (25), 20143–20167. 10.1039/d5ra01014d 40519680 PMC12163905

[B9] Gao S. Li X. Jiang Q. Liang Q. Zhang F. Li S. (2022). PKM2 promotes pulmonary fibrosis by stabilizing TGF-β1 receptor I and enhancing TGF-β1 signaling. Sci. Adv. 8, eabo0987. 10.1126/sciadv.abo0987 36129984 PMC9491720

[B10] Hao M. Guan Z. Zhang Z. Ai H. Peng X. Zhou H. (2023). Atractylodinol prevents pulmonary fibrosis through inhibiting TGF-β receptor 1 recycling by stabilizing vimentin. Mol. Ther. 31, 3015–3033. 10.1016/j.ymthe.2023.08.017 37641404 PMC10556230

[B11] Hisatomi K. Mukae H. Sakamoto N. Ishimatsu Y. Kakugawa T. Hara S. (2012). Pirfenidone inhibits TGF-β1-induced over-expression of collagen type I and heat shock protein 47 in A549 cells. BMC Pulmonary Medicine 12, 24. 10.1186/1471-2466-12-24 22694981 PMC3403980

[B12] Huang Y. Wang C. Zhou T. Xie F. Liu Z. Xu H. (2024). Lumican promotes calcific aortic valve disease through H3 histone lactylation. Eur. Heart J. 45, 3871–3885. 10.1093/eurheartj/ehae407 38976370

[B13] Jiang Y. Gu J. Niu X. Hu J. Zhang Y. Li D. (2024). Phosphorylation-regulated dynamic phase separation of HIP-55 protects against heart failure. Circulation 150, 938–951. 10.1161/CIRCULATIONAHA.123.067519 38328928 PMC11404759

[B14] Karunarathne W. A. H. M. Lee K. T. Choi Y. H. Kang C. H. Lee M. H. Kim S. H. (2024). Investigating rutin as a potential transforming growth factor-β type I receptor antagonist for the inhibition of bleomycin-induced lung fibrosis. BioFactors 50, 477–492. 10.1002/biof.2020 38006284

[B15] Khirallah S. M. Ramadan H. M. M. Aladl H. A. A. Ayaz N. O. Kurdi L. A. F. Jaremko M. (2022). Antidiabetic potential of novel 1,3,5-trisubstituted-2-thioxoimidazloidin-4-one analogues: insights into α-glucosidase, α-amylase, and antioxidant activities. Pharmaceuticals 15, 1576. 10.3390/ph15121576 36559028 PMC9785777

[B16] Lee Y. Rio D. C. (2015). Mechanisms and regulation of alternative Pre-mRNA splicing. Annu. Rev. Biochem. 84, 291–323. 10.1146/annurev-biochem-060614-034316 25784052 PMC4526142

[B17] Li J. Xiao Y. Zhang Y. Li S. Zhao M. Xia T. (2023). Pulmonary delivery of specialized pro-resolving mediators-based nanotherapeutics attenuates pulmonary fibrosis in preclinical animal models. ACS Nano 17, 15354–15370. 10.1021/acsnano.2c10388 37535431

[B18] Li C. Feng X. Li S. He X. Luo Z. Cheng X. (2024). Tetrahedral DNA loaded siCCR2 restrains M1 macrophage polarization to ameliorate pulmonary fibrosis in chemoradiation-induced murine model. Mol. Ther. 32, 766–782. 10.1016/j.ymthe.2024.01.022 38273656 PMC10928155

[B19] Li D. Zhang X. Song Z. Zhao S. Huang Y. Qian W. (2024). Advances in common *in vitro* cellular models of pulmonary fibrosis. Immunol. Cell. Biol. 102, 557–569. 10.1111/imcb.12756 38714318

[B20] Li F. Li X. Dai S. Yang Z. Bao Z. Wang S. (2024). Efficient light-based bioprinting via rutin nanoparticle photoinhibitor for advanced biomedical applications. ACS Nano 18, 22104–22121. 10.1021/acsnano.4c05380 39102149

[B21] Li X. Chen M. Chen X. He X. Li X. Wei H. (2024). TRAP1 drives smooth muscle cell senescence and promotes atherosclerosis via HDAC3-primed histone H4 lysine 12 lactylation. Eur. Heart J. 45, 4219–4235. 10.1093/eurheartj/ehae379 39088352 PMC11481199

[B22] Li B. Li X. H. Zhou Y. Feng D. D. Luo Z. Q. Cheng H. P. (2026). Lactylation in pulmonary fibrosis: current understanding and challenges. J. Transl. Med. 24, 514. 10.1186/s12967-026-07991-9 41803869 PMC13085443

[B23] Licheva M. Babic R. Pflaum J. Mancilla H. Wilfling F. Kraft C. (2025). To degrade or not to degrade: how phase separation modulates selective autophagy. Autophagy 12, 1–3. 10.1080/15548627.2025.2476025 40052694 PMC12674472

[B24] Liu C. Wei W. Huang Y. Fu P. Zhang L. Zhao Y. (2024). Metabolic reprogramming in septic acute kidney injury: pathogenesis and therapeutic implications. Metabolism 158, 155974. 10.1016/j.metabol.2024.155974 38996912

[B25] Liu H. Xu Q. Wufuer H. Li Z. Sun R. Jiang Z. (2024). Rutin is a potent senomorphic agent to target senescent cells and can improve chemotherapeutic efficacy. Aging Cell 23 (1), e13921. 10.1111/acel.13921 37475161 PMC10776113

[B26] Luo C. Huang C. Zhu Y. Zhou Y. Qiao Y. Shi C. (2025). Inhibition of Rho GEFs attenuates pulmonary fibrosis through suppressing myofibroblast activation and reprogramming profibrotic macrophages. Cell. Death Dis. 16 (1), 278. 10.1038/s41419-025-07573-5 40216763 PMC11992128

[B27] Mao Q. Liu J. Yan Y. Wang G. Zhang M. Wang Z. (2025). 13-Methylpalmatine alleviates bleomycin-induced pulmonary fibrosis by suppressing the ITGA5/TGF-β/Smad signaling pathway. Phytomedicine 140, 156545. 10.1016/j.phymed.2025.156545 40023972

[B28] Mohamed D. I. Abo Nahas H. H. Elshaer A. M. El-Waseef D. A. E. A. El-Kharashi O. A. Mohamed S. M. Y. (2023). Unveiling the interplay between NSAID-Induced dysbiosis and autoimmune liver disease in children: insights into the hidden gateway to autism spectrum disorders. Evidence from *ex vivo*, *in vivo,*, and clinical studies. Front. Cell. Neurosci. 17, 1268126. 10.3389/fncel.2023.1268126 38026692 PMC10644687

[B29] Peng Y. Mei S. Qi X. Tang R. Yang W. Feng J. (2025). PGC-1α mediates migrasome secretion accelerating macrophage-myofibroblast transition and contributing to sepsis-associated pulmonary fibrosis. Exp. Mole. Med 57, 759–774. 10.1038/s12276-025-01426-z 40164683 PMC12046055

[B30] Pergolizzi J. V. Jr. LeQuang J. A. Varrassi M. Breve F. Magnusson P. Varrassi G. (2023). What do we need to know about rising rates of idiopathic pulmonary fibrosis? A narrative review and update. Adv. Ther. 40, 1334–1346. 10.1007/s12325-022-02395-9 36692679 PMC9872080

[B31] Qiu Q. Deng H. Song P. Liu Y. Zhang M. (2025). Lactylation in glioblastoma: a novel epigenetic modifier bridging epigenetic plasticity and metabolic reprogramming. Int. J. Mol. Sci. 26 (7), 3368. 10.3390/ijms26073368 40244246 PMC11989911

[B32] Raghu G. Fleming T. R. (2024). Moving forward in IPF: lessons learned from clinical trials. Lancet Respir. Med. 12, 583–585. 10.1016/S2213-2600(24)00177-2 38880115

[B33] Rangarajan S. Kurundkar A. Kurundkar D. Bernard K. Sanders Y. Y. Ding Q. (2016). Novel mechanisms for the antifibrotic action of nintedanib. Am. J. Res. Cell. Mole. Bio 54, 51–59. 10.1165/rcmb.2014-0445OC 26072676 PMC4742925

[B34] Wang M. Yang H. Wang Y. Zhao Y. Kang D. Song X. (2026). Blocking the phase separation of hnRNP L attenuates pulmonary fibrogenesis by inhibiting fibroblast-to-myofibroblast differentiation. Curr. Gene Ther. 10.2174/0115665232425283251206012409 42613704

[B35] Wang Y. Hu R. Zhang X. Cai G. Ding Q. Che P. (2026). PKM2 mediates the glycolytic response to matrix stiffness in lung fibroblasts. Front. Cell. Dev. Biol. 14, 1771078. 10.3389/fcell.2026.1771078 42148308 PMC13176260

[B36] Xu P. Zhang J. Wang M. Liu B. Li R. Li H. (2022). hnRNPL-activated circANKRD42 back-splicing and circANKRD42-mediated crosstalk of mechanical stiffness and biochemical signal in lung fibrosis. Mol. Therapy 30, 2370–2387. 10.1016/j.ymthe.2022.01.045 35278674 PMC9171245

[B37] Yang J. Huang Y. Cui Z. Liu C. Xie G. (2024). Rhizoma coptidis can inhibit the excessive proliferation, inflammation, and transformation of lung fibroblasts into myofibroblasts. Allergol. Immunopathol. Madr. 52, 15–20. 10.15586/aei.v52i4.1111 38970260

[B38] Yang Y. Wang X. Zhang J. (2024). Pirfenidone and nintedanib attenuate pulmonary fibrosis in mice by inhibiting the expression of JAK2. J. Thoracic Disease 16, 1128–1140. 10.21037/jtd-23-1057 38505034 PMC10944717

[B39] Zhou X. Zou L. Liao H. Luo J. Yang T. Wu J. (2022). Abrogation of hnRNP L enhances anti-PD-1 therapy efficacy *via* diminishing PD-L1 and promoting CD8+ T cell-mediated ferroptosis in castration-resistant prostate cancer. Acta Pharm. Sin. B 12, 692–707. 10.1016/j.apsb.2021.07.016 35256940 PMC8897216

