# Supplementary material for: Rutin interferes with hnRNP L phase separation and modulates the PKM2–lactate–H3K9la axis in pulmonary fibrosis
Source: Front Pharmacol. 2026 Oct 16;17:1787570. doi: 10.3389/fphar.2026.1787570 (PMC13719308; doi:10.3389/fphar.2026.1787570)
Supplement: Supplementary file 1 [file Supplementaryfile1.docx]

**Supporting information**

**Rutin inhibits PKM2-lactate-H3K9la-PKM2 feedback loop by suppressing hnRNP L phase separation to alleviate pulmonary fibrosis**

**Jianlin Luan^1#^, Yujie Wang^1#^, Mengqi Jiang^1^, Haitong Zhang^3^, Changjun Lv^3^, Jinjin Zhang^1*^, Xiaodong Song^1*^**

1 School of Pharmacy, Shandong Key Lab of Complex Medical Intelligence and Aging, Shandong Medical and Pharmaceutical University, Yantai, China,

2 Department of Respiratory and Critical Care Medicine, Shandong Medical and Pharmaceutical University Hospital, Shandong Medical and Pharmaceutical University, Binzhou, China

**Correspondence:**

Jinjin Zhang and Xiaodong Song, School of Pharmacy, [Shandong Medical and Pharmaceutical University](https://baike.baidu.com/item/%E5%B1%B1%E4%B8%9C%E5%8C%BB%E8%8D%AF%E5%A4%A7%E5%AD%A6/67434480). No. 346, Guanghai Road, Yantai 264003, China.

Email: [jjinzhang@126.com](mailto:jjinzhang@126.com); songxd71@sdmpu.edu.cn

Tel: +86-0535-6913985; Fax: +86-0535-6913985

**#These authors contributed equally to this work.**

Figure S1: (A) Western blot showing the effect of rutin on H3K9la and α‑SMA expression in TGF‑β1‑induced cells with or without lactic acid.

Figure S2: (A) Types of alternative splicing and the number of corresponding events following hnRNP L knockdown and the proportion of variable splicing events with significant differences (B) KEGG pathway enrichment analysis identified 12 signaling pathways enriched by genes corresponding to differential alternative splicing events under hnRNP L regulation. (C) A heatmap of the top 50 differential splicing events.

Figure S3: qRT-PCR analysis of α-SMA mRNA expression in TGF-β1-stimulated IMR-90 cells. Cells were transfected with hnRNP L overexpression plasmid, hnRNP L-∆1–111/∆274–384 overexpression plasmid, or negative control (NC), followed by treatment with or without rutin.
